# Dryland Cropping Systems, Weed Communities, and Disease Status Modulate the Effect of Climate Conditions on Wheat Soil Bacterial Communities

**DOI:** 10.1128/mSphere.00340-20

**Published:** 2020-07-15

**Authors:** Suzanne L. Ishaq, Tim Seipel, Carl Yeoman, Fabian D. Menalled

**Affiliations:** a University of Oregon, Biology and the Built Environment Center, Eugene, Oregon, USA; b Montana State University, Department of Land Resources and Environmental Sciences, Bozeman, Montana, USA; c Montana State University, Department of Animal and Range Sciences, Bozeman, Montana, USA; University of Illinois at Urbana-Champaign

**Keywords:** 16S rRNA gene, Illumina MiSeq, climate change, conventional, grazing, organic, tillage, wheat streak mosaic virus

## Abstract

Little knowledge exists on how soil bacteria in agricultural settings are impacted by management practices and environmental conditions in current and predicted climate scenarios. We assessed the impact of soil moisture, soil temperature, weed communities, and disease status on soil bacterial communities in three cropping systems: (i) conventional no-till (CNT) systems utilizing synthetic pesticides and herbicides, (ii) USDA-certified tilled organic (OT) systems, and (iii) USDA-certified organic systems with sheep grazing (OG). Sampling date within the growing season and associated soil temperature and moisture exerted the greatest effect on bacterial communities, followed by cropping system, Wheat streak mosaic virus (WSMV) infection status, and weed community. Soil temperature was negatively correlated with bacterial richness and evenness, while soil moisture was positively correlated with bacterial richness and evenness. Soil temperature and soil moisture independently altered soil bacterial community similarity between treatments. Inoculation of wheat with WSMV altered the associated soil bacteria, and there were interactions between disease status and cropping system, sampling date, and climate conditions, indicating the effect of multiple stressors on bacterial communities in soil. In May and July, cropping system altered the effect of climate change on the bacterial community composition in hotter conditions and in hotter and drier conditions compared to ambient conditions, in samples not treated with WSMV. Overall, this study indicates that predicted climate modifications as well as biological stressors play a fundamental role in the impact of cropping systems on soil bacterial communities.

**IMPORTANCE** Climate change is affecting global moisture and temperature patterns, and its impacts are predicted to worsen over time, posing progressively larger threats to food production. In the Northern Great Plains of the United States, climate change is forecast to increase temperature and decrease precipitation during the summer, and it is expected to negatively affect cereal crop production and pest management. In this study, temperature, soil moisture, weed communities, and disease status had interactive effects with cropping system on bacterial communities. As local climates continue to shift, the dynamics of above- and belowground associated biodiversity will also shift, which will impact food production and increase the need for more sustainable practices.

## Abstract (precis)

Climate change is affecting global moisture and temperature patterns, and its impacts are predicted to worsen over time, posing progressively larger threats to food production. In the Northern Great Plains of the United States, climate change is forecast to increase temperature and decrease precipitation during the summer, and it is expected to negatively affect cereal crop production and pest management. In this study, temperature, soil moisture, weed communities, and disease status had interactive effects with cropping system on bacterial communities. As local climates continue to shift, the dynamics of above- and belowground associated biodiversity will also shift, which will impact food production and increase the need for more sustainable practices.

## INTRODUCTION

Climate change affects soil moisture content and temperature, which, in turn, impacts crop production and nutritional value (1–4); pest abundance, dynamics, and management (4–7); and overall ecosystem resiliency (8). Determining how climate change modifies multitrophic interactions between crops, weeds, pathogens, and soil microbial communities is complex (9), yet critical, as crop production relies on healthy soil and microbially mediated nutrient cycling (10, 11). Microbial α-diversity in soil is linked to plant growth stage (12). Low microbial α-diversity in soil is associated with impeded plant growth and early senescence of Arabidopsis thaliana (13). With the knowledge that climate change will fundamentally change the dynamics of agricultural ecosystems, we must increase our understanding of the mechanisms driving biological and environment stress to secure the sustainability of agricultural production (1, 14, 15).

The Northern Great Plains of the United States is a major global cereal-producing region where the effects of climate change are already being felt (16, 17). Over the next 30 years, mean temperature is predicted to increase by 2.5 to 3.3°C in this region (17, 18). Soil microbial community structure and function may be altered due to their temperature sensitivity (19–21). This temperature increase coupled with predicted decreases in summer precipitation will yield hotter and drier conditions during the growing season, resulting in crop stress (17), which has the potential to further alter soil microbial communities. In periods of drought, microbial diversity is reduced (22, 23), as is the ability of the microbes to cycle soil nitrogen (24). Drought can also cause plants to prioritize relationships with fungi over bacteria, reducing the transfer of nutrients and contributing to the crash of the bacterial community (25, 26). Further, as climate change alters the composition of plant communities and their nutrient content (27; T. Seipel, S. L. Ishaq, and F. D. Menalled, submitted for publication), the composition of plant litter and residues is altered. This change in soil inputs, in turn, modifies plant-microbe relationships (28–30) and reduces the available nutrients recycled into soil (22, 29).

Climate change is also predicted to worsen the effects of plant pathogens, including *Wheat streak mosaic virus* (WSMV; genus *Tritimovirus*), either by altering the dynamics of vector transfer or by decreasing plant resistance to infection (7, 31). WSMV is transmitted by wheat curl mites (*Aceria tosichella*), occurs across the North American Great Plains, and can make plants more susceptible to the effects of climate change by hindering root development and water uptake (32). To our knowledge, no study has formally assessed the potential link between WSMV infection and plant-, rhizosphere-, or root-associated microbial communities. It is possible that WSMV infections alter root structure or function and can alter the capacity for plants to interact with soil microbiota.

In industrial (contemporarily referred to as conventional) cropping systems, management approaches focused on maximizing production are based on regular applications of synthetic inputs in the form of fertilizers and pesticides (33). In recent years, shifted consumer demands and new market opportunities have developed organic production into a major agricultural, economic, and cultural force (34, 35). However, organic cropping systems rely heavily on tillage for weed management and cover-crop termination. Due to the negative consequences that tilling has on the physical, chemical, and biological properties of soils in the semiarid ecosystems that dominate large sections of the Northern Great Plains, there is a growing interest among farmers and researchers in reducing soil disturbance practices in organic systems (36–38). In this context, the integration of crop and livestock production has been proposed as a sustainable approach to terminate cover crops, manage crop residues, and control weeds while reducing tillage intensity (39–41); however, very few studies exist on the impact of integrated livestock management on soil quality or microbial communities (23) or disease resistance.

Differences among cropping systems affect plant communities, including species abundance, composition, and growth (42, 43), which, in turn, modifies microbial communities in the rhizosphere (23, 44, 45). Although previous studies have evaluated the role of microbial communities in crop yields and crop-weed competition (46), fewer have explored the extent to which root-associated bacteria are impacted by cropping systems, weeds, and plant disease in current and predicted climate scenarios. The aim of our study was to assess changes in soil bacterial communities due to warmer and drier climate conditions and the presence of WSMV across contrasting cropping systems and their associated weed communities. We hypothesized that (i) bacterial community richness and evenness would be reduced by climate or WSMV infection; (ii) cropping systems that promote bacterial richness would be more resistant to alterations from climate and WSMV infection; and (iii) more diverse bacterial communities would have a more stable bacterial community membership over the growing season and in response to increased soil temperature, decreased precipitation, and WSMV.

## RESULTS

### Bacterial diversity and evenness.

**FIG 1:**
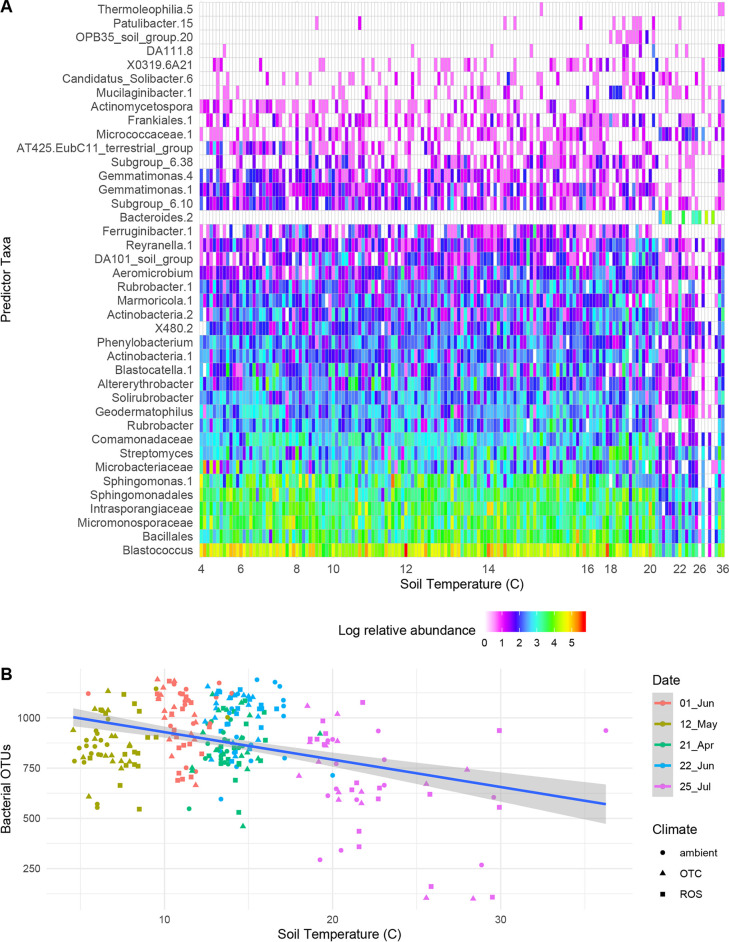
Effect of soil temperature on soil bacterial communities. (A) Soil temperature was negatively correlated with soil bacterial richness. (B) Relative abundance of soil bacterial by temperature over the 2016 growing season, selected as important features by random forest classification. Taxa are arranged by total relative abundance, and only statistically significant features are shown. The model explained 45% of variance.

Soil temperature during the growing season (see Fig. S1 in the supplemental material) was a strong driver of bacterial species richness (Table 1), with fewer bacterial operational taxonomic units (OTUs; 97% cutoff) observed in soil during hotter temperatures (Fig. 1A). Increased soil temperature reduced the evenness of bacterial species (Table 1). Higher soil temperatures were negatively associated with the presence or relative abundance of bacterial taxa that were significantly important features in the model (Fig. 1B). The most abundant of those taxa included members of *Blastococcus*, *Bacillales*, *Micromonosporaceae*, *Intrasporangiaceae*, *Sphingomonas*, *Microbacteriaceae*, and *Streptomyces* (Fig. 1B). There were 42 OTUs which were significantly associated with a climate treatment (Table 2); 11 were associated with ambient conditions, 17 with hotter conditions, and 14 with hotter and drier conditions.

**TABLE 1:** Effect of treatment factors and their interactions on observed soil bacterial richness and evenness

Factor or interaction	Observed richness^*a*^			Shannon evenness		
	Sum of squares	*F* value	*P* value	Sum of squares	*F* value	*P* value
Cropping system (C)	275,240	4.53	0.012	0.051	6.28	0.002
Soil temperature (T)	2,513,973	82.82	<0.001	0.080	19.64	<0.001
Soil moisture (M)	1,201,709	39.59	<0.001	0.028	6.97	0.009
WSMV (V)	56,697	1.87	0.173	0.001	0.14	0.708
Date^*b*^	2,203,758	28.71	<0.001	0.104	8.6	<0.001
C:T	442,913	7.30	0.001	0.072	8.84	<0.001
C:M	217,218	3.58	0.029	0.015	1.89	0.154
T:M	845,614	27.86	<0.001	0.025	6.07	0.014
C:V	110,178	1.81	0.165	0.001	0.17	0.844
T:V	49,186	1.62	0.204	0.001	0.32	0.571
M:V	23,029	0.76	0.385	0.001	0.17	0.683
C:T:M	179,405	2.96	0.054	0.010	1.24	0.293
C:T:V	59,576	0.98	0.376	0.008	0.96	0.385
C:M:V	11,219	0.18	0.831	0.002	0.21	0.812
T:M:V	4,413	0.15	0.703	0.001	0.37	0.546
C:T:M:V	8.038	0.13	0.876	0.005	0.59	0.556

a Richness is measured as bacterial taxon counts and evenness of taxon abundance on a scale from 0 to 1 (each species having equal abundance). Comparisons were made using a linear mixed-effects model accounting for repeated measures of subplots within replicated blocks, and significance was determined via type III ANOVA with Satterthwaite's approximation.

b Factor used in simple model.

**TABLE 2:** Bacteria taxa significantly affected by climate treatment

Condition(s)	OTU	LDA	*P* value^*a*^
Ambient	*Agromyces*	2.19	0.013
	*Betaproteobacteria*	2.18	0.026
	*Cytophagaceae*	2.29	0.0122
	*Gaiella*	2.44	0.006
	*Gemmatimonadaceae*	2.28	<0.001
	*Chloroflexi* KD4-96	2.49	0.033
	*Chloroflexi* KD4-96	2.63	0.003
	*Betaproteobacteria* subgroup 6	2.13	0.040
	*Sphingomonadales*	2.86	0.020
	*Acidobacteria* subgroup 6	2.15	0.021
	*Acidobacteria* subgroup 6	2.39	0.044

OTC; hotter	*Agromyces*	2.25	<0.001
	*Gemmatimonadaceae*	2.32	0.012
	*Microbacteriaceae*	2.92	0.010
	*Myxococcales*	2.17	0.008
	*Oxalobacteraceae*	2.79	0.001
	*Ramlibacter*	2.14	0.003
	*Segetibacter*	2.30	0.001
	*Acidobacteria* subgroup 6	2.00	0.010
	*Acidobacteria* subgroup 6	2.15	0.024
	*Acidobacteria* subgroup 6	2.24	<0.001
	*Acidobacteria* subgroup 6	2.26	0.018
	*Acidobacteria* subgroup 6	2.30	0.018
	*Acidobacteria* subgroup 6	2.31	0.011
	*Acidobacteria* subgroup 6	2.36	<0.001
	*Acidobacteria* subgroup 6	2.36	0.009
	*Acidobacteria* subgroup 6	2.52	0.019
	*Xanthomonadaceae*	2.54	0.025

ROS; hotter and drier	*Holophagae* ABS-19	2.33	0.023
	*Amycolatopsis*	2.34	0.003
	*Comamonadaceae*	2.13	0.017
	*Conexibacter*	2.20	0.018
	*Kineosporia*	2.74	<0.001
	*Mycobacterium*	2.59	0.018
	*Nocardioides*	2.19	0.003
	*Rhizobium*	2.34	0.007
	*Sandaracinaceae*	2.20	0.010
	*Sphingomonas*	2.59	0.015
	*Sphingomonas*	2.69	0.032
	*Streptomyces*	3.01	0.004
	*Acidobacteria* subgroup 6	2.40	0.004
	*Xanthomonadaceae*	2.43	0.028

a Significance was determined by LEFSE with an LDA cutoff score of >2.

10.1128/mSphere.00340-20.5 FIG S1 Soil temperatures during the 2016 growing season at the Fort Ellis Research and Teaching Center in Bozeman, MT. Download FIG S1, TIF file, 0.3 MB. Copyright © 2020 Ishaq et al. 2020 Ishaq et al. This content is distributed under the terms of the Creative Commons Attribution 4.0 International license.

**FIG 2:**
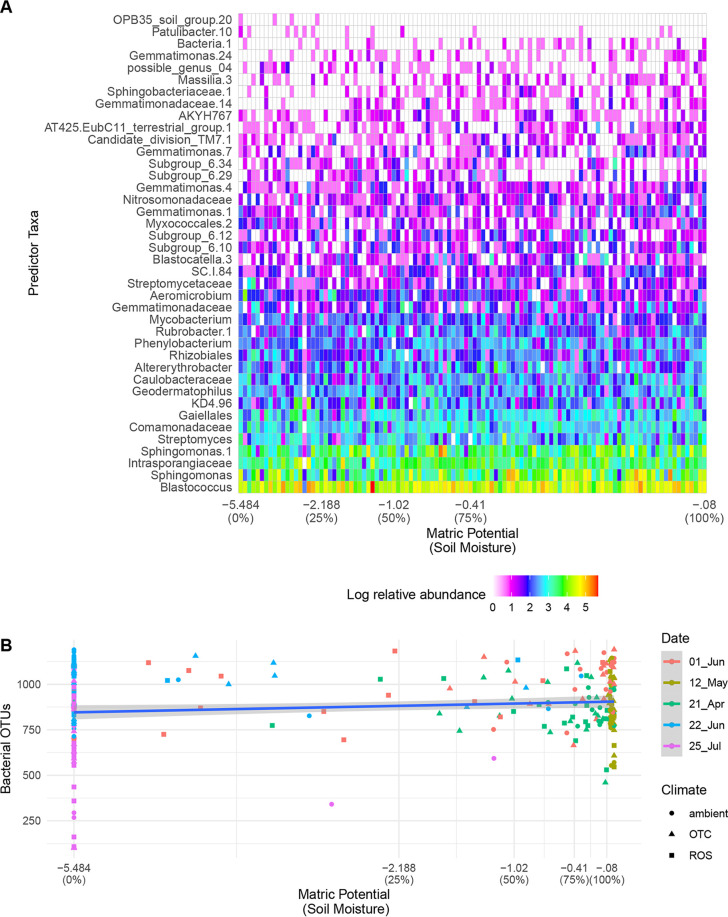
Effect of soil moisture on soil bacterial communities. (A) Soil moisture was positively correlated with soil bacterial richness. (B) Relative abundances of rhizosphere bacteria were affected by soil moisture from all subplots across the 2016 growing season, selected as important features by random forest classification (*P < *0.05). The model explained 32% of variance. Soil moisture is presented as matric potential on the main *x* axis and percent saturation on the secondary *x* axis.

Soil moisture during the growing season (Fig. S2) positively impacted total bacterial species richness (Fig. 2A; Table 1) and evenness (Table 1), though not as strongly as temperature did. Across all samples, soil temperature and soil moisture were not correlated with each other (lmer, *P > *0.05) (Fig. S3). Soil moisture impacted the relative abundance of bacterial species in different ways (Fig. 2B). For example, *Aeromicrobium* organisms were more abundant at low soil moisture levels, *Sphingomonas* organisms were more abundant at high moisture, and *Phenylobacterium* organisms were most abundant at moderate levels of soil moisture (Fig. 2B).

10.1128/mSphere.00340-20.6 FIG S2 Soil moisture during the 2016 growing season at the Fort Ellis Research and Teaching Center in Bozeman, MT. Cropping systems include chemical no-till (Conv), organic grazed (GrazeO), and organic tilled (TillOrg). Download FIG S2, TIF file, 0.2 MB. Copyright © 2020 Ishaq et al. 2020 Ishaq et al. This content is distributed under the terms of the Creative Commons Attribution 4.0 International license.

10.1128/mSphere.00340-20.7 FIG S3 Soil temperature was not correlated with soil moisture over the 2016 growing season, lmer, *P > *0.05. Download FIG S3, TIF file, 0.2 MB. Copyright © 2020 Ishaq et al. 2020 Ishaq et al. This content is distributed under the terms of the Creative Commons Attribution 4.0 International license.

**FIG 3:**
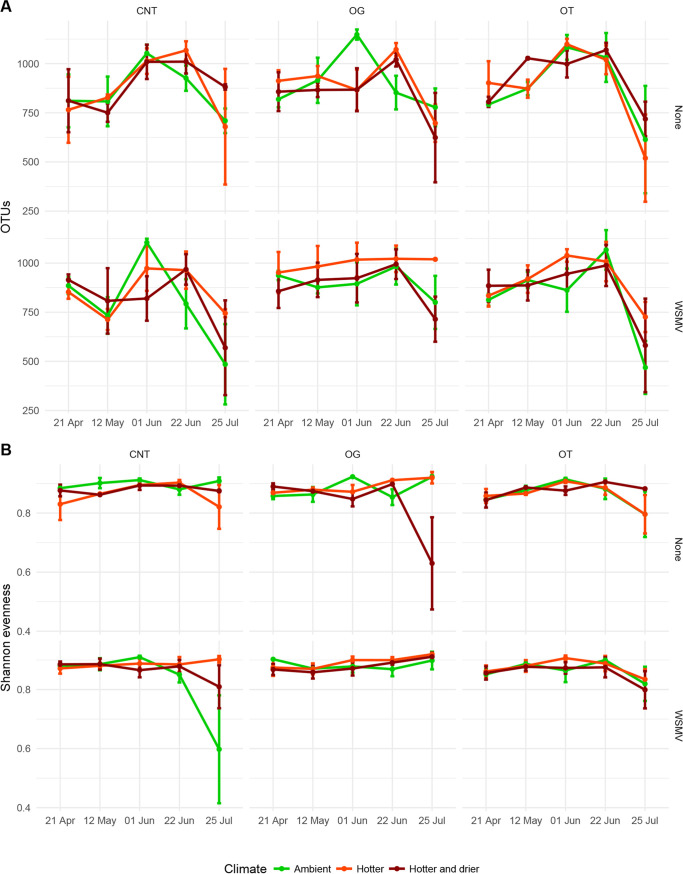
Soil bacterial richness and evenness over the 2016 growing season. (A) Species-level richness and (B) species-level evenness by cropping system (conventional no-till [CNT], organic grazed [OG]. and organic tilled [OT]), climate conditions (ambient, hotter, and hotter and drier), and pathogen infection (Wheat streak mosaic virus [WSMV] or a no-template control [none]). Error bars show standard errors of the means (SEM).

Cropping system (conventional no-till [CNT], organic grazed [OG], or organic tilled [OT]) interacted with climate treatment to affect bacterial richness and evenness (Fig. 3; Table 1). Bacterial richness under ambient conditions peaked in early June for all three cropping systems, while richness under both hotter conditions and hotter and drier conditions peaked in late June in the CNT and OG systems (Fig. 3A). Bacterial richness in OG subplots was affected by soil temperature (lmer, estimate = 35, *F* = 2.997, *P = *0.003) and moisture (lmer, estimate = 6, *F* = 2.203, *P = *0.03). There were 344 OTUs which were significantly associated with a climate treatment (Table S1); 106 were associated with the CNT system, 170 with the OG system, and 68 with the OT system.

10.1128/mSphere.00340-20.1 TABLE S1 Bacteria taxa significant to cropping system. Significance was determined by LEFSE with an LDA cutoff score of >2. Download Table S1, DOCX file, 0.04 MB. Copyright © 2020 Ishaq et al. 2020 Ishaq et al. This content is distributed under the terms of the Creative Commons Attribution 4.0 International license.

Inoculation with WSMV resulted in 6 positive CNT samples with a mean infection rate within subplots of 4.4%, 9 positive OG samples with a mean infection rate of 13.3%, and 7 positive OT samples with a mean infection rate of 3.2% (Table S2). Overall, inoculation with WSMV had no effect on bacterial species richness or evenness (Fig. 3; Table 1). When CNT and OT subplots were compared, there was a date-virus interaction on bacterial richness (lmer, *F* = 2.6792, *P = *0.039). OT subplots at the end of July that had been inoculated with WSMV showed reduced bacterial richness (lmer, *F* = 2.019, *P = *0.046), as did all hotter subplots in July treated with WSMV (*F* = 3.046, *P = *0.003) and hotter OG subplots inoculated with WSMV in April (*F* = 2.039, *P = *0.044), May (*F* = 2.088 *P = *0.039), and late June (*F* = 2.192, *P = *0.03). Weed species diversity and percent coverage or biomass did not alter bacterial richness across all subplots (lm, *P > *0.05). There was one OTU significantly associated with subplots not treated with virus (*Solirubrobacterales* 288-2, linear discriminant analysis [LDA] = 2.01, *P = *0.037) and 3 OTUs with WSMV inoculation (*Acetobacteraceae*, LDA = 2.42, *P = *0.006; *Bacillales*, LDA = 2.03, *P = *0.046; and *Flavobacterium*, LDA = 3.08, *P = *0.020).

10.1128/mSphere.00340-20.2 TABLE S2 Wheat streak mosaic virus infection rates in July 2016. Cropping systems include chemical no-till (CNT), organic grazed (OG), and organic tilled (OT). Virus treatment includes Wheat streak mosaic virus (WSMV) and no-template control (none). Climate treatments include ambient conditions, hotter (OTC), and hotter and drier (ROS). Download Table S2, DOCX file, 0.03 MB. Copyright © 2020 Ishaq et al. 2020 Ishaq et al. This content is distributed under the terms of the Creative Commons Attribution 4.0 International license.

### Bacterial community stability.

Soil temperature impacted bacterial community similarity with significant but relatively weak effects (Table 3). Higher soil temperatures were associated with increased variation in bacterial community heterogeneity and dispersion (betadisper, *F* = 3.3579, *P < *0.001); i.e., in warmer temperatures the bacterial communities were more dissimilar across and within a treatment group. Soil moisture also weakly but significantly altered soil bacterial community similarity (Table 3) but did not affect the amount of variation (heterogeneity) within bacterial communities (betadisper, *P > *0.05). Soil moisture did not have an effect on homogeneity when healthy and WSMV subplots were considered separately, to account for the effect of WSMV on plants’ abilities to take up water. Soil bacterial community similarity was minorly impacted by the interaction of cropping system and climate (Table 3).

**TABLE 3:** PERMANOVA of treatment factors and their interactions on soil bacterial communities^*a*^

Factor or interaction	Bray-Curtis			
	*F* value	*R* ^2^	*P* value	Significance^*b*^
Cropping system (C)	0.88	0.007	0.001	***
Soil moisture (M)	3.67	0.014	0.001	***
Soil temperature (T)	2.00	0.007	0.004	**
Virus (V)	1.63	0.006	0.001	***
Date^*c*^	5.27	0.077	<0.001	***
C:M	1.15	0.008	0.213	
C:T	1.69	0.012	0.005	**
M:T	2.88	0.011	0.001	***
C:V	1.00	0.007	0.189	
M:V	1.82	0.007	0.015	*
T:V	2.93	0.011	0.001	***
C:M:T	1.03	0.008	0.411	
C:M:V	1.56	0.011	0.009	**
C:T:V	1.25	0.009	0.107	
M:T:V	2.22	0.008	0.001	***
C:M:T:V	1.25	0.009	0.093	t

a Comparisons were made accounting for repeated measures of subplots and with replicate blocks as a stratification.

b ***, <0.001; **, 0.001 to 0.009; *, 0.01 to 0.05; t (trending), 0.05 to 0.1.

c Factor used in simple model.

**FIG 4:**
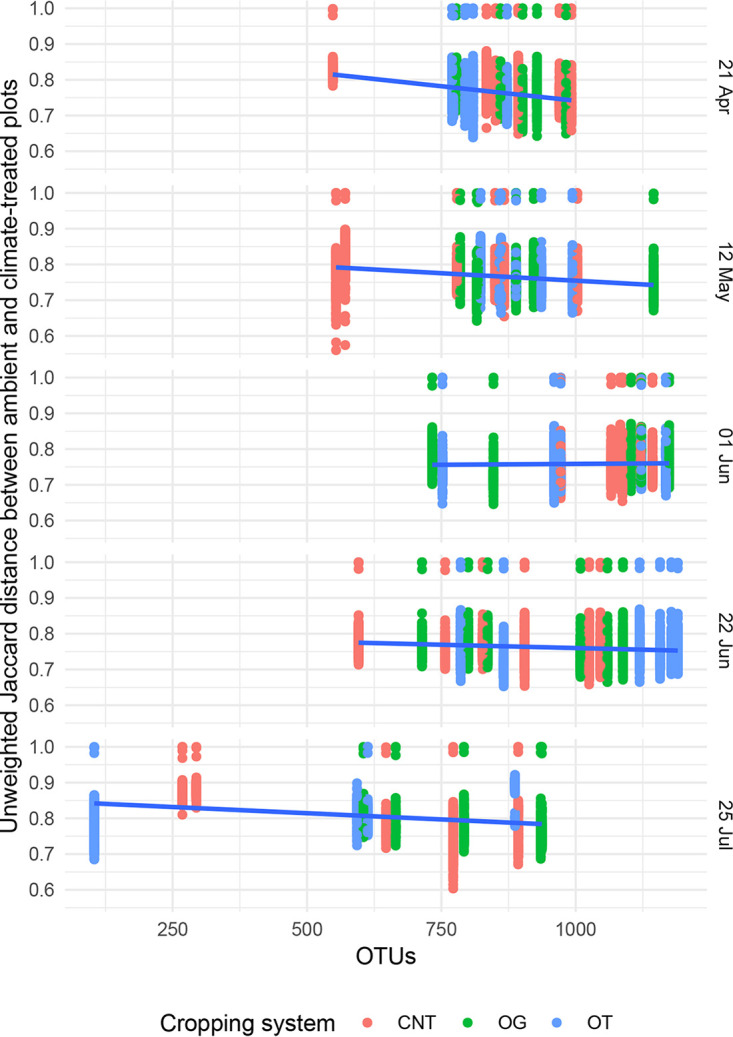
Soil bacterial community similarity between ambient and climate-treated subplots correlated with bacterial OTUs. Cropping systems include conventional no-till (CNT), organic grazed (OG), and organic tilled (OT).

**FIG 5:**
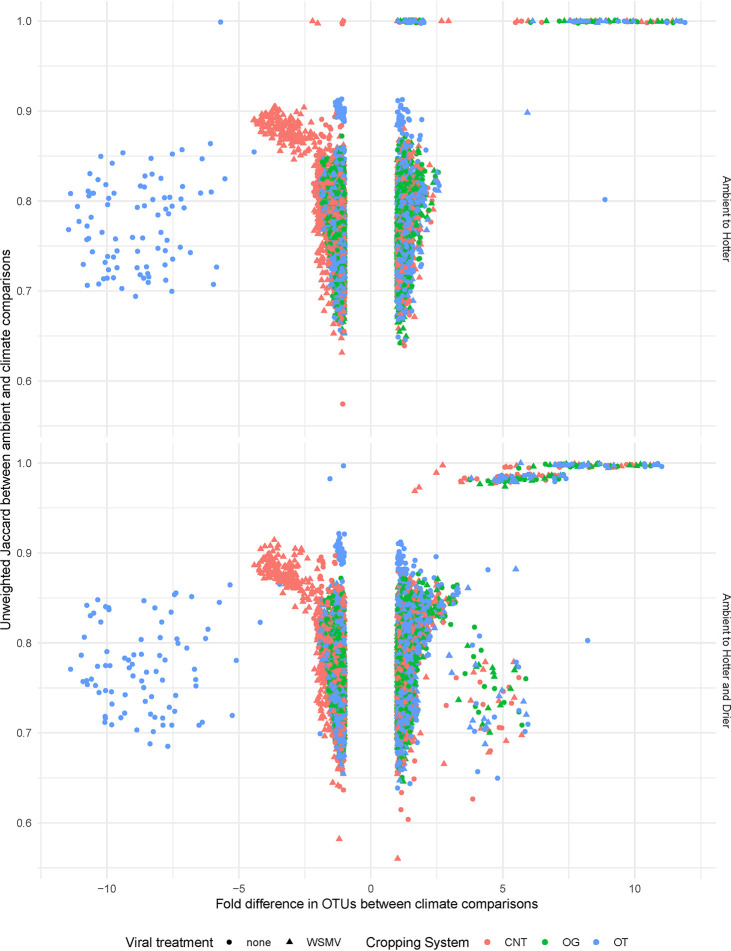
Soil bacterial community similarity against the fold change in number of OTUs in comparing ambient to hotter subplots and ambient to hotter and drier subplots across the 2016 growing season. The difference in OTUs is measured as fold change, or ratio of the OTU abundance in ambient subplots over the OTU abundance in climate scenario subplots. Viral treatment includes Wheat streak mosaic virus (WSMV) and a no-template control (none). Cropping systems include conventional no-till (CNT), organic grazed (OG), and organic tilled (OT).

Here, stability (interpreted as no significant difference in bacterial β-diversity) was similar between ambient and treatment subplots over time. Lower OTU richness correlated with a higher similarity between ambient and manipulated hotter and drier subplots (lm, *F* = 1291, *P* < 0.001), and this was most evident early and late in the growing season (Fig. 4). The temporal stability of a bacterial community against climate change was not associated with a lower fold difference in OTUs between ambient and hotter subplots and ambient and hotter and drier subplots (Fig. 5). While the most stable soil communities did have more bacterial OTUs, a loss of OTUs was not necessarily associated with having lower community similarity (Fig. 5).

**FIG 6:**
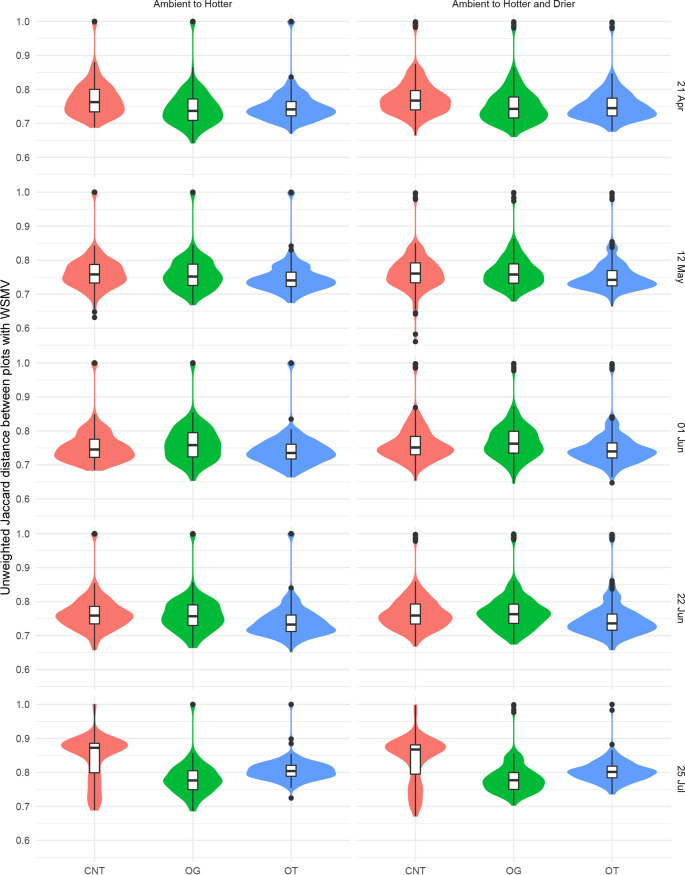
Soil bacterial community similarity between ambient and hotter and between ambient and hotter and drier conditions in subplots from three cropping systems across the 2016 growing season. Plots were not treated with Wheat streak mosaic virus. Significance is provided in Table 4. Cropping systems include conventional no-till (CNT), organic grazed (OG), and organic tilled (OT).

When bacterial communities’ responses to climate conditions were compared, cropping system affected how stable bacterial communities remained at different periods in the growing season (Fig. 6; Table 4). In May, the bacterial communities in hotter OG samples were less stable than the ambient OG samples, in contrast to those in hotter CNT and OT samples, which were more stable than the corresponding bacterial communities under ambient conditions (Fig. 6; Table 4). In July, the bacterial communities in the hotter OT subplots were more stable than those in the hotter CNT or OT subplots and the respective ambient conditions (Fig. 6; Table 4). For bacterial communities in hotter and drier subplots compared to ambient subplots, communities in OT subplots showed the most stability, followed by OG samples, and CNT were least stable (most dissimilar) compared to the respective ambient conditions (Fig. 6; Table 4).

**TABLE 4:** Effect of climate conditions, cropping system, and sampling date on soil bacterial community composition^*a*^

Comparison	Cropping system^*b*^	Date	Virus^*c*^	Adjusted *P* value
Ambient vs. hotter	OG-CNT	12 May	None	0.002
	OT-OG	12 May	None	0.001
	OT-CNT	25 Jul	None	<0.001
	OT-OG	25 Jul	None	<0.001
	OG-CNT	21 Apr	WSMV	<0.001
	OT-CNT	21 Apr	WSMV	0.001
	OT-OG	1 Jun	WSMV	0.032
	OT-CNT	22 Jun	WSMV	<0.001
	OT-OG	22 Jun	WSMV	0.007
	OG-CNT	25 Jul	WSMV	<0.001
	OT-CNT	25 Jul	WSMV	<0.001
	OT-OG	25 Jul	WSMV	0.003

Ambient vs. hotter and drier	OG-CNT	25 Jul	None	0.006
	OT-CNT	25 Jul	None	<0.001
	OT-OG	25 Jul	None	<0.001
	OG-CNT	21 Apr	WSMV	0.001
	OT-CNT	21 Apr	WSMV	0.002
	OT-OG	22 Jun	WSMV	0.001
	OT-CNT	22 Jun	WSMV	0.007
	OG-CNT	25 Jul	WSMV	<0.001
	OT-CNT	25 Jul	WSMV	<0.001

a The unweighted Jaccard index was used to calculate bacterial community composition, and comparisons were made between ambient and hotter conditions and between ambient and hotter and drier conditions within each sampling date. Comparisons were tested with analysis of variance, and *P* values were adjusted with Tukey’s honestly significant differences.

b Cropping systems: CNT, conventional no-till; OG, organic grazed; OT, organic tilled.

c WSMV, Wheat streak mosaic virus.

**FIG 7:**
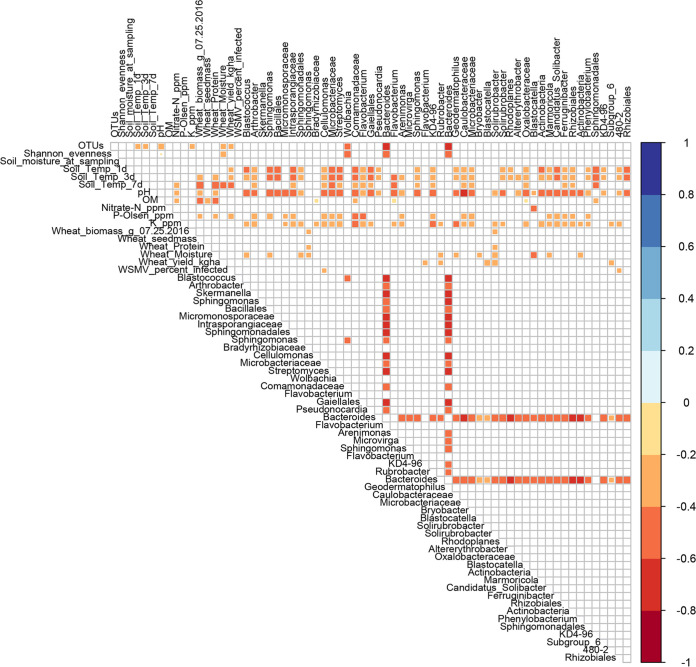
Spearman’s correlations between most abundant soil bacteria and various factors at the end of the 2016 growing season in July. Significant correlations are shown, determined by Wilcoxon rank (*P < *0.05).

**FIG 8:**
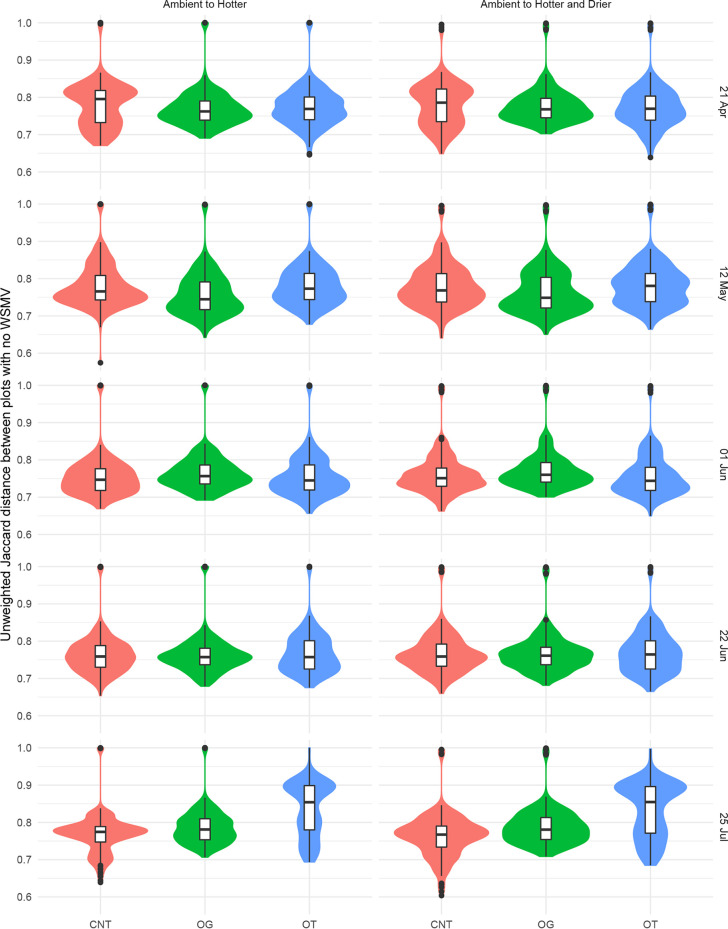
Soil bacterial community similarity between ambient and hotter conditions and between ambient and hotter and drier conditions in subplots treated with Wheat streak mosaic virus from three cropping systems across the 2016 growing season. Significance is provided in Table 4. Cropping systems include conventional no-till (CNT), organic grazed (OG), and organic tilled (OT).

Neither WSMV inoculation nor rate of infection (Table S2) within subplots created a definable bacterial community (random forest; data not shown), although WSMV inoculation was negatively associated with a species of *Cellulomonas*, as well as with *Actinobacteria* clade 480-2 (Fig. 7). However, WSMV inoculation significantly affected soil bacterial community similarity (Table 3). Moreover, there was an interaction of WSMV and climate change (Table 3), which was modulated by cropping system (Fig. 8; Table 4).

When the changes in bacterial communities between ambient conditions and hotter conditions or hotter and drier climate conditions were compared, cropping system modulated how stable bacterial communities remained in subplots which had been treated with WSMV. In assessing similarity between bacterial communities between ambient and climate conditions, CNT and OT subplots treated with WSMV were significantly different (analysis of variance [ANOVA], *P < *0.001 [Tukey’s test]) from their noninfected counterparts, indicating that disease status altered the ability of the community to remain stable (i.e., resistance) under changing climate. However, OG subplots did not differ between WSMV-treated and untreated subplots (ANOVA, *P > *0.05 [Tukey’s test]) in terms of the similarity between ambient and climate-conditioned soil.

WSMV made it more difficult for bacterial communities to remain stable under different climate conditions, across the growing season, and between cropping systems (Fig. 8; Table 4). When ambient conditions were compared to hotter conditions in subplots treated with WSMV, in April, CNT subplots were more stable than OG or OT subplots; in early June, OG subplots were more stable than OT subplots; in late June, CNT and OG subplots were more stable than OT subplots; and in late July, CNT subplots were most stable, followed by OT and then OG subplots (Fig. 8; Table 4). A comparison of hotter and drier conditions to ambient conditions in WSMV-treated subplots showed that in April, CNT subplots were more stable than OG or OT subplots; in late June, CNT and OG subplots were more stable than OT subplots; and in late July, CNT subplots were again more stable than OG or OT subplots (Fig. 6; Table 4).

Weed communities in the organic systems were more diverse than the CNT subplots, though OG and CNT subplots had similar relative species abundance (Seipel et al., submitted). Climate conditions had minor impacts on weed communities (Seipel et al., submitted). Weed species diversity, as well as percent biomass from subplots, negatively impacted the similarity between ambient and climate-treated subplots across the growing season (Fig. S4), including weed diversity (lm, *F* = 79.153, *P < *0.001) and percent coverage (*F* = 26.516, *P < *0.001) the prior fall on 25 October 2015, diversity (*F* = 25.637, *P* < 0.001) and coverage (*F* = 119.78, *P* < 0.001) early in the growing season on 8 April 2016, diversity on 14 June 2016 (*F* = 68.888, *P* < 0.001), and weed biomass on 29 June 2016 (*F* = 30.807, *P* < 0.001).

10.1128/mSphere.00340-20.8 FIG S4 Soil bacterial community distance between ambient and climate-treated subplots, in response to weed community dynamics in the 2016 growing season at the Fort Ellis Research and Teaching Center in Bozeman, MT. Cropping systems include chemical no-till (CNT), organic grazed (OG), and organic tilled (OT). Download FIG S4, TIF file, 0.5 MB. Copyright © 2020 Ishaq et al. 2020 Ishaq et al. This content is distributed under the terms of the Creative Commons Attribution 4.0 International license.

**FIG 9:**
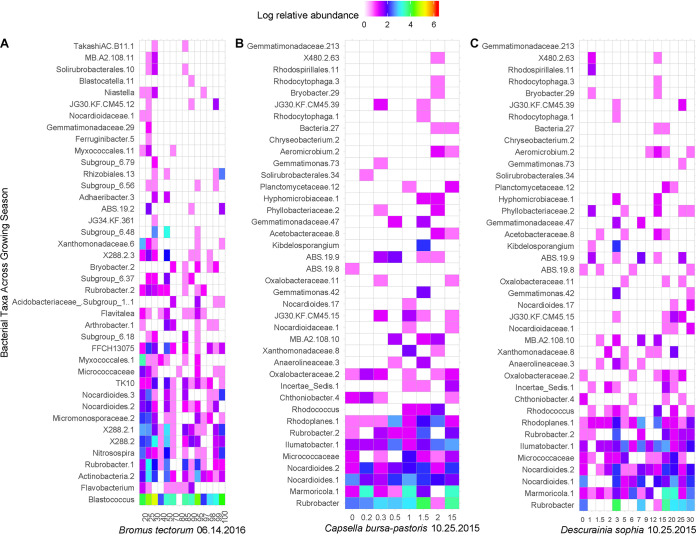
Relative abundance of rhizosphere bacteria associated with *Bromus tectorum* (A), *Capsella bursa-pastoris* (B), and *Descurainia sophia* (C) subplot coverage at specific points during the 2016 growing season. Bacterial taxa were selected as important features by random forest classification (*P < *0.05).

Individual weed species were weakly but significantly correlated with membership of the soil bacterial community (Table 5), including *Asperugo procumbens*, *Bromus tectorum*, *Capsella bursa-pastoris*, *Chenopodium album*, *Cirsium arvense*, *Descurainia sophia*, *Galium aparine*, *Lactuca serriola*, *Lamium amplexicaule*, *Malva neglecta*, *Monolepsis nuttalliana*, *Poa annua*, *Solanum triflorum*, *Taraxacum officinale*, *Thlaspi arvense*, *Tragopogon dubius*, and *Trifolium pretense.* Of these, three winter annuals had definable effects on bacterial community structure (Fig. 9) (*P < *0.05). *Bromus tectorum* cover in mid-June (Fig. 9A), as well as cover of *Capsella bursa-pastoris* (Fig. 9B) and *Descurainia sophia* (Fig. 9C) in the previous fall, had a predictable impact on the rhizosphere community. *Bromus tectorum* had a U-shaped relationship with bacterial relative abundance, while *C. bursa-pastoris* and *D. sophia* showed more of a positive correlation. *Capsella bursa-pastoris* cover in subplots associated with an increase in *Rubrobacter*, *Nocardioides*, *Ilumatobacter*, the family-level clade FFCH13075 in the order *Solirubrobacterales*, and others (Fig. 9B). *Descurainia sophia* coverage of subplots was associated with an increase in the KD4-96 clade in the phylum *Chloroflexi*, FFCH13075, *Blastococcus*, *Nocardioides*, *Oryzihumus*, and others (Fig. 9C).

**TABLE 5:** PERMANOVA of weed species identity and percent coverage on soil bacterial communities at different times over a growing season^*a*^

Weed and measurement	*F* model	*R* ^2^	*P* value	Significance^*b*^
*Asperugo procumbens*, cov, Oct 2015	1.212	0.00474	0.047	*
*Bromus tectorum*, cov, Oct 2015	0.819	0.0032	0.001	***
*Bromus tectorum*, cov, Jun 2016	1.016	0.00397	0.005	**
*Bromus tectorum*, biomass, late Jun 2016	1.1495	0.00449	0.001	***
*Capsella bursa-pastoris*, cov, Oct 2015	0.7862	0.00307	0.035	*
*Capsella bursa-pastoris*, cov, Apr 2016	1.0965	0.00429	0.001	***
*Capsella bursa-pastoris*, biomass, late Jun 2016	1.0236	0.004	0.001	***
*Chenopodium album*, cov, Apr 2016	1.1424	0.00447	0.05	*
*Chenopodium album*, cov, Jun 2016	1.0397	0.00407	0.001	***
*Cirsium arvense*, biomass, late Jun 2016	0.8499	0.00332	0.03	*
*Descurainia sophia*, cov, Oct 2015	0.7804	0.00305	0.003	**
*Galium aparine*, cov, Apr 2016	0.7757	0.00303	0.049	*
*Lactuca serriola*, biomass, late Jun 2016	0.9691	0.00379	0.042	*
*Lamium amplexicaule*, cov, Oct 2015	1.0349	0.00405	0.047	*
*Malva neglecta*, cov, Oct 2015	1.129	0.00441	0.019	*
*Malva neglecta*, cov, Apr 2016	0.6295	0.00246	0.009	**
*Monolepsis nuttalliana*, cov, Jun 2016	0.9573	0.00374	0.009	**
*Poa annua*, cov, Oct 2015	0.8321	0.00325	0.001	***
*Poa annua*, cov, Apr 2016	0.7457	0.00292	0.022	*
*Solanum triflorum*, cov, Oct 2015	0.8559	0.00335	0.044	*
*Taraxacum officinale*, cov, Oct 2015	0.8523	0.00333	0.002	**
*Taraxacum officinale*, cov, Jun 2016	0.7226	0.00283	0.021	*
*Thlaspi arvense*, cov, Apr 2016	1.3834	0.00541	0.002	**
*Thlaspi arvense*, cov, Jun 2016	0.8235	0.00322	0.047	*
*Tragopogon dubius*, biomass, late Jun 2016	0.799	0.00312	0.002	**
*Trifolium pratense*, biomass, late Jun 2016	0.8271	0.00323	0.001	***
*Trifolium pratense*, cov, Apr 2016	0.813	0.00318	0.024	*

a Comparisons were made accounting for repeated measures of subplots and with replicate blocks as a stratification. Only significant comparisons are shown. cov, coverage.

b ***, <0.001; **, 0.001 to 0.009; *, 0.01 to 0.05.

## DISCUSSION

This study evaluated the effects of climate conditions, WSMV inoculation, cropping system, and associated *in situ* weed communities on wheat soil bacterial communities over the course of a growing season. We hypothesized that (i) bacterial community richness and evenness would be reduced by climate changes or WSMV infection, (ii) cropping systems which promote bacterial richness would be more resistant to alterations from climate changes and WSMV infection, and (iii) more diverse bacterial communities would have a more stable bacterial community membership over the growing season and in response to increased soil temperature, decreased precipitation, and WSMV. In summary, sampling date within the growing season, soil temperature, and soil moisture exerted the greatest effect on soil bacterial communities, followed by cropping system, WSMV infection status, and weed community characteristics.

Changes in precipitation and soil moisture, atmospheric gas concentration, soil salinity, and soil temperature can affect bacterial diversity (19, 20, 47). In particular, soil temperature can be a stronger driver of bacterial diversity and functionality than soil moisture (48). Even after weeks of warmer temperatures, soil microbiotas do not appear to develop functional resistance to the heat and maintain stable communities (48), yet soils which experience frequent wet-dry cycles, such as grassland soils, host microbial communities which remain more stable under drought conditions (49). Physical or chemical disturbance can further prevent a stable soil community which is adapted to warmer temperatures from forming (50). In this study, and in accordance with previous studies (51, 52), soil temperature was found to be a stronger driver of bacterial species diversity and abundance than soil moisture. This may reflect the more complex interaction between plants, microorganisms, and soil conditions, as soil moisture can somewhat stabilize soil temperature (53). Plant foliation, which increases with air temperature, in turn shades soil and can buffer further increases in soil temperature (53), thus better supporting microbial communities.

Cropping systems are known to be associated with particular soil microbial communities (23, 45, 54). In particular, the use of plant- or manure-based fertilizer can increase microbial diversity, while chemical-based fertilizers select for acid-tolerant species (45, 55, 56), leading to the trend of organic systems to harbor more diverse microbial communities than conventional (industrial) ones (55, 57, 58). Further, tillage and herbicides reduce microbial diversity (59, 60). Soil microbial α-diversity has been used as a topical application to rescue plants from drought, salt stress, or disease (61–63) and may be used to remediate soils after chemical or physical disruption (64–67). Thus, management practices which promote microbial diversity have the potential to be used as an *in situ* method to moderate the effect of stressors such as climate change, pathogens, or weeds (46, 54).

We previously assessed the impact of these cropping systems over the course of the growing season (23) and observed that under ambient conditions, cropping system did not alter bacterial richness or evenness but did affect β-diversity. In particular, organic tilled subplots contained more putative nitrogen-fixing bacterial genera (23). In the present study, the bacterial community in all cropping systems changed over the course of the growing season, as well as in response to increased soil temperature or decreased soil moisture. However, the interaction between cropping systems and climate conditions was not identical across systems. The peak in bacterial richness in CNT and OG systems was delayed in the hotter and the hotter and drier conditions compared to their respective ambient subplots. From observations, wheat in these systems developed more slowly than in OT subplots. The peak in bacterial richness is likely tied to peak growth and development of wheat, when plant-bacterial nutrient exchange is greatest. In OT subplots, the earlier peak and subsequent drop in bacterial richness may be associated with a more advanced growth stage and earlier senescence (13). Cropping system affected the stability of bacterial communities when ambient conditions were compared to climate-treated conditions, with the conventional no-till system remaining more stable than the organic ones. This may reflect the more intense selective pressure exerted by chemical inputs on the community and the recruitment of a more resilient microbiota.

Cropping system can indirectly alter soil microbial α-diversity via crop disease susceptibility. For example, direct nitrogen fertilization can increase WSMV disease transmission (68). Using livestock grazing to terminate cover crops and control weed residues can reduce wheat mite populations (69), although this has not been shown to reduce virus transmission (70). In the present study, there were interactions between WSMV application and soil moisture, soil temperature, and cropping system-soil moisture, pointing to the importance of multiple concurrent stressors in shaping soil communities. The effect of different cropping systems on viral infection in crops is complex (71) and is largely modulated by the extent of crop diversification, crop residue removal strategy, and pest control (72).

As early successional species, agricultural weeds establish quickly in newly disturbed soil and sometimes earlier in the growing season than spring or summer crops (73). In climate change scenarios which predict warmer, wetter springs and higher atmospheric CO_2_, the alteration of the local environmental conditions can give weeds a greater advantage over crops (74). Changing environmental conditions and crop-weed competition may, in turn, alter the soil microbial community, further making conditions less favorable for crop germination, growth, and competitive ability (75). As with all plant species, agricultural weed species associate with particular microbial communities in their rhizosphere (23, 54, 73, 76). It is generally thought that weed diversity in agricultural settings could increase microbial diversity in soil and potentially increase the functionality and stability of soil microbial communities. In this study site, ambient subplots were previously shown to have weak positive correlations between weed diversity and soil bacterial richness (23). In the present study, weed diversity or biomass did not alter soil bacterial richness or evenness, although bacterial β-diversity was affected, and weed diversity was inversely related to the stability of bacterial communities in response to climate treatment. This may reflect the temporary increases in bacterial richness during periods of weed growth which are not sustained during the hottest part of the season when bacterial communities are more susceptible to temperature and moisture stress.

The effects of environmental condition or disease status had interactions with cropping system when bacterial communities were assessed. This has implications for soil bacterial communities and plant performance (77), both within the growing season and in successive plantings, as the legacy of these altered bacterial communities persists (8). As local climates continue to shift, so too will the dynamics of above- and belowground diversity, which will impact food production and drive the need for more sustainable practices (5, 16, 18).

### Conclusions.

Overall, this study indicates that predicted climate modifications as well as biological stressors play a fundamental role in the impact of cropping systems on soil bacterial communities. Soil temperature, soil moisture, treatment with Wheat streak mosaic virus, type of cropping system, and date within the growing season were shown to have independent and interacting effects on soil bacterial community richness, evenness, and stability over time.

## MATERIALS AND METHODS

### Experimental design.

This study was conducted in 2015 and 2016 at an agricultural field experiment that had been implemented since July 2012 at the Montana State University Fort Ellis Research and Teaching Center, Bozeman, MT (45.652664056 N, −110.97249611 W; elevation, 1,500 m above sea level), to test production of three dryland cropping systems using a 5-year crop rotation. The Fort Ellis site is a Blackmore silt loam soil type (a fine-silty, mixed, superactive, frigid Typic Arguistoll) with a consistent ratio of 1 part sand, 2 parts silt, and 1 part clay, by weight, at 0% to 4% slopes (78). The monthly air temperature in Bozeman in 2016 was higher than historic maxima and minima from 1981 to 2010, and the mean monthly precipitation (Table S3; republished from *Geoderma* [23]) was lower by 18 mm in May, 16 mm in June, and 14 mm in July (79).

10.1128/mSphere.00340-20.3 TABLE S3 Temperature and precipitation in Bozeman, MT, during the 2016 growing season and 1981–2010 growing seasons. (Republished from *Geoderma* [23].) Download Table S3, DOCX file, 0.02 MB. Copyright © 2020 Ishaq et al. 2020 Ishaq et al. This content is distributed under the terms of the Creative Commons Attribution 4.0 International license.

The cropping systems at the studied site consisted of (i) a conventional no-till system (CNT), in which synthetic inputs were used in the form of fertilizers, herbicides, and fungicides; (ii) a USDA-certified tilled organic (OT) system, and (iii) a USDA-certified organic system with grazing (OG), which integrates sheep grazing to terminate cover crops and manage weeds, with the overall goal of reducing tillage intensity in organic production. Chemical inputs utilized in the CNT system included 2,4-dichorophenoxyacetic acid (2,4-D), bromoxynil, dicamba, fluroxypyr, glyphosate, 2-methyl-4-chlorophenoxyacetic acid, pinoxaden, and urea for winter wheat rotations (see Tables 2.7 and 2.8 in reference 80). The organic plots began the organic transition process in July 2012 and completed it in 2015. In the OT system, tillage was performed with a chisel plow, tandem disk, or field cultivator, as needed to control weeds, prepare the seedbed, and incorporate cover crops and crop residues. Weed control was enhanced with a rotary harrow. In the OG system, targeted sheep grazing was used to reduce tillage intensity for preseeding and postharvest weed control and to terminate the cover crops, with duration and intensity of grazing based on weed biomass (5). Grazing was minimally supplemented with tillage, based on soil conditions and weed pressure. For all systems, seeding was done with a low-disturbance no-till double-disk seeder. Outside normal farm management activities, soil disturbance and compaction were minimized during sampling procedures. Further details of the management practices, both historical and at the time of experimentation, can be found elsewhere (5, 42, 80).

Each system was replicated three times (i.e., blocks) with cropping systems (75 by 90 m) as the main plots, each of which was further divided into 5 split plots (13 by 90 m), with a 2-m fallow buffer between. Split plots were each following a 5-year rotation, as follows: year 1, safflower (*Carthamus tinctorius* L.) undersown to yellow sweet clover [*Melilotus officinalis* (L.) Lam.]; year 2, sweet clover cover crop; year 3, winter wheat (Triticum aestivum L.); year 4, lentil (*Lens culinaris* Medik.); and year 5, winter wheat (5).

Within each of the year 3 (winter wheat) fields, subplots (1-m diameter) were randomly established to assess the impact of climate conditions and disease status on wheat soil bacteria across cropping systems. Two subplots were marked with flags and used as control or ambient climate conditions (ambient conditions), two subplots were enclosed with an open-top chamber (OTC; hotter conditions) made from 18-inch-high plastic that reflected heat back on the subplot to increase air temperature and soil temperature by 1 to 2°C (81), and two subplots were enclosed with OTCs and partially covered with rain-out shelters (OTC-ROS; hotter and drier conditions) which reduced rainfall by 50% using transparent polyurethane (Fig. S5; similar to conditions described in reference 82). For each of the three climate treatments, one of the subplots was randomly inoculated with WSMV (see below).

10.1128/mSphere.00340-20.9 FIG S5 Example of climate chamber treatments. Left to right: ambient conditions; increased temperature (hotter) with open-top chambers (OTCs); increased temperature and decreased rainfall (hotter and drier) with open-top chambers and rain-out shelters (ROS). Download FIG S5, TIF file, 2.1 MB. Copyright © 2020 Ishaq et al. 2020 Ishaq et al. This content is distributed under the terms of the Creative Commons Attribution 4.0 International license.

### Wheat streak mosaic virus inoculation and data collection.

Following previous work (83), prior to the WSMV inoculations, spring wheat (variety Chouteau) was grown in the greenhouse in flat trays (30 by 10 cm), where plants were maintained under a 16-h photoperiod of sunlight supplemented with mercury vapor lamps (165 μE m^−2^ s^−1^) at 10°C/25°C (day/night). When the wheat was at Feekes stage 4 to 6, an inoculum of WSMV was created from the Conrad isolate line (84). Infected wheat was harvested from the greenhouse and frozen for 1 to 2 days until use. To create the WSMV inoculum, 300 g of infected wheat clippings were ground to reduce particle size using a food processor and then blended with buffer (3.2 liters of deionized water plus 600 ml of 5× phosphate-buffered solution, pH 7.2) until smooth. The slurry was filtered through cheesecloth to remove particulate matter which would clog the spray hose and refrigerated for up to 1 h until use. Immediately prior to use, 2 g carborundum (ground glass) was added per 3.78 liters of slurry as an abrasive to injure wheat slightly but enough for the virus to infect it. Slurry was sprayed onto subplots using an air compressor (275 kPa) travelling at a rate of 0.5 m/s and sprayed at a height of 20 cm above the canopy. Control subplots were sprayed with water in which 2 g carborundum was added per 3.78 liters (no-template control). Spraying occurred the last week of April, 1 week after the first soil sampling date (21 April), and 2 weeks prior to the second sampling date (12 May).

Infection of WSMV in subplots was evaluated in July by using an indirect enzyme-linked immunosorbent assay (ELISA), with 10 leaves sampled from each subplot and assessed separately (85). Within a plate, every 10th well contained a negative control (i.e., a sample from a healthy wheat plant) to reduce potential bias in values of optical density caused by position of samples. The mean and standard deviation for the negative control on each plate were calculated. Samples with values above three standard deviations were considered infected with WSMV (86). ELISA results are provided in Table S1.

### Crop and weed evaluations.

Percent coverage of weeds in subplots was assessed visually in October 2015, April 2016, and June 2016. Aboveground biomass of all weed species within sampled areas was harvested by hand in late June 2016. Within each 0.75-m^2^ subplot, weed biomass was cut at ground level and separated by species. The individual biomass of each species was dried for 2 weeks at 55°C and weighed (Seipel et al., submitted). Wheat biomass was harvested from sampled areas by hand on 25 July 2016, once the crop had completely senesced and ripened. The two center rows (75 cm each) of wheat in the subplot were harvested, for a total of 1.5 row-meters. All the aboveground biomass was harvested, dried for 1 week at 55°C, and threshed to determine biomass and grain yields (Seipel et al., submitted).

### Soil assessment.

Soil moisture was measured weekly using gypsum blocks buried 5 cm belowground (87). Percent moisture readings below 0 were outside the range of measurement and were reset to 0, per manufacturer recommendations; then matric potential was calculated according to previous literature (88). Soil temperature was measured with buried iButtons (Maxim Integrated), with data obtained every 4 h between 14 April 2016 (1 week prior to the first sampling) and 25 July 2016 (final sampling date). In each subplot, three cores were taken from around wheat plants to a depth of 15 cm and then homogenized into one composite sample, which was used for bacterial community sampling (stored at −20°C) and nutrient analysis (stored at 4°C). Soil cores were obtained from all 54 subplots at five time points over the growing season: 21 April, before the WSMV inoculations were applied; 12 May, 1 week post-WSMV infection; 1 June, 3 weeks post-WSMV infection; 22 June, 6 weeks post-WSMV infection; and 25 July, 10 weeks post-WSMV infection and immediately prior to wheat harvesting. Additional soil was collected at wheat harvest for nutrient analysis (Table S4) (Agvise Laboratories, Northwood, ND, USA).

10.1128/mSphere.00340-20.4 TABLE S4 Soil properties measured at wheat harvest (25 July 2016). Values are means with standard deviations in parentheses. Superscripts represent significant paired interactions (*P < *0.05). Cropping system included chemical no-till (CNT), organic tilled (OT), and organic grazed (OG). Viral treatment included Wheat streak mosaic virus (WSMV) or no-template control (none). Download Table S4, DOCX file, 0.02 MB. Copyright © 2020 Ishaq et al. 2020 Ishaq et al. This content is distributed under the terms of the Creative Commons Attribution 4.0 International license.

DNA extraction from soil samples (PowerSoil 96-well soil DNA isolation kit; MoBio Laboratories, Inc.), library preparation (HotStart PCR kit; Kapa Biosystems), sequencing, and sequence analysis protocols were as previously described (23). An Illumina MiSeq system (Montana State University, Bozeman, MT) was used to sequence the V3-V4 region of the 16S rRNA gene, using primers 341F (5′-CCTACGGGAGGCAGCAG-3′) and 806R (5′-GGACTACHVGGGTWTCTAAT-3′) (89).

The bioinformatics workflow, described in more detail elsewhere (23), used PANDAseq (90) to assemble contigs, mothur version 1.38 (91) to process quality control steps, and R version 3.5 (92) to perform statistical analysis. Significant taxa by treatment class were assessed using the mothur-integrated version of linear discriminant analysis effect size (LEFSe) (93) with an LDA cutoff score of >2.

Linear mixed-effects models and distance-based redundancy models (vegan) (94), random forest with permutation (95, 96), permutational ANOVA (PERMANOVA; adonis) (97), and ggplot2 (98) were used in the R statistical package (92). Linear mixed models used cropping system, soil moisture, and soil temperature on the day of sampling and WSMV application as random effects. Sampling date and subplot identity nested with block were included to control for repeated sampling. Some variables were aliased in the distance-based redundancy analysis and therefore were removed from the model: *Capsella bursa-pastori*s, *Cirsium arvense*, *Galium aparine*, and *Tragopogon dubius* biomass on 29 June 2016; *Chenopodium album*, *Lamium amplexicaule*, *Malva neglecta*, *Poa annua*, and *Solanum triflorum* coverage on 25 October 2015; *C. arvense*, *T. dubius*, and *Trifolium pretense* coverage on 8 April 2016; and *Chenopodium album* and *Tragopogon dubius* coverage on 14 June 2016. Plant coverage has been shown to have a linear correlation with plant aboveground biomass (99, 100), and weed senescence may negate the effect on soil microorganisms (100). Thus, as coverage was measured at multiple time points but biomass only once, coverage was used as a more accurate measure of the weed-soil microbe relationship with respect to sampling date when coverage and biomass were both significant.

Random forest was performed with 500 trees and 100 permutations. Replicate block did not affect numerical diversity and was included as a random effect in those models, but it did affect bacterial communities when ambient systems were compared (23) and was included as a fixed effect in those models. Unweighted Jaccard similarity was used to determine effect of factors on community structure and tested with permutational analysis of variance (PERMANOVA; adonis function), with replicate block as a stratification. When comparing climate to ambient conditions, we utilized analysis of variance (ANOVA) and Tukey’s honestly significant differences to assess the variables determining soil bacterial communities. The comparison and visualization of ambient and modified climate conditions was based on R code developed by Ashkaan Fahimipour and Roo Vandegrift.

### Data availability.

Sequencing output data can be found in the Sequence Read Archive (SRA) at NCBI under BioProject no. PRJNA383161.
