# Supplementary figures and images for: Dryland Cropping Systems, Weed Communities, and Disease Status Modulate the Effect of Climate Conditions on Wheat Soil Bacterial Communities
Source: mSphere. 2020 Jul 15;5(4):e00340-20. doi: 10.1128/mSphere.00340-20 (PMC7364210; doi:10.1128/mSphere.00340-20)

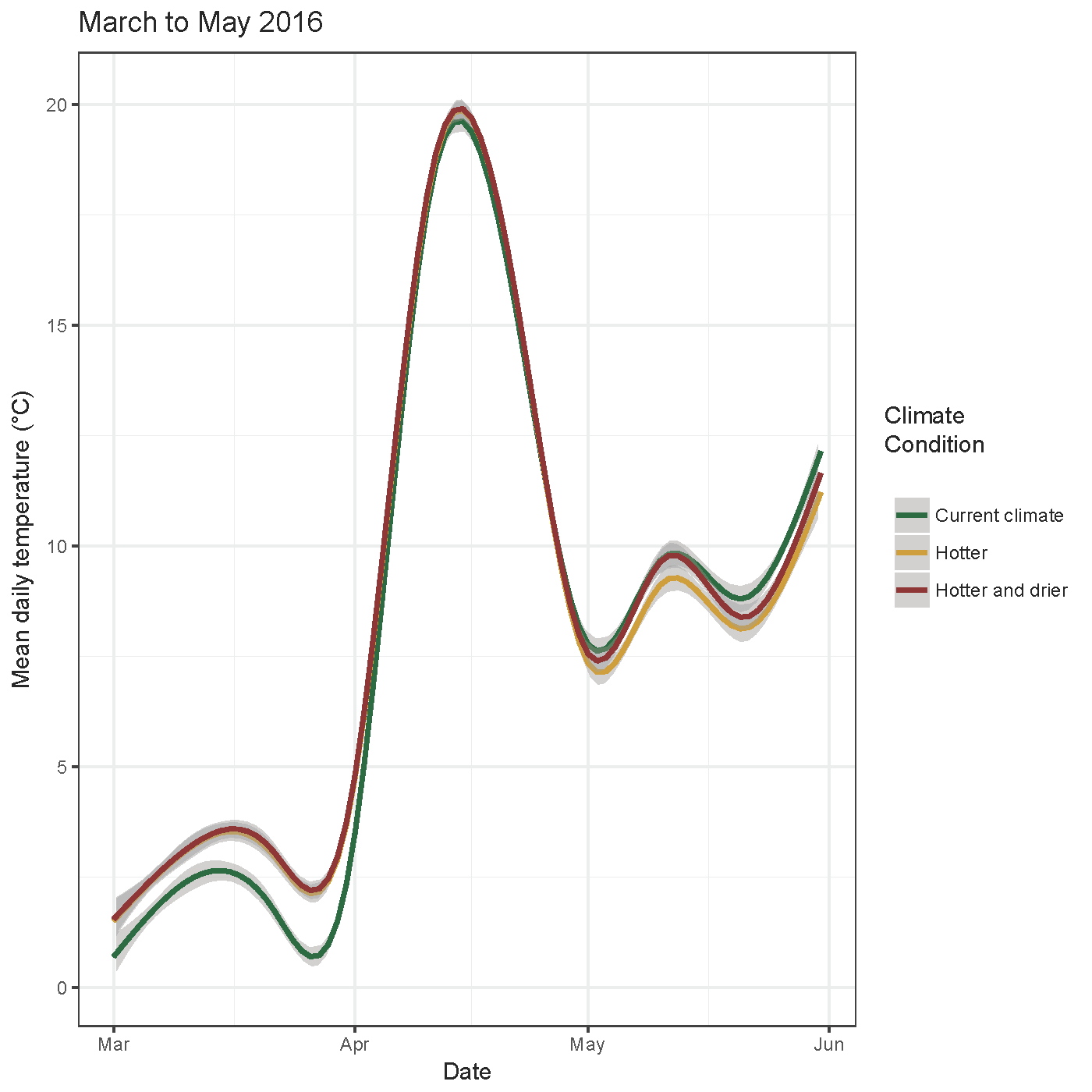

Supplement: FIG S1 [file mSphere.00340-20-sf001.tif]

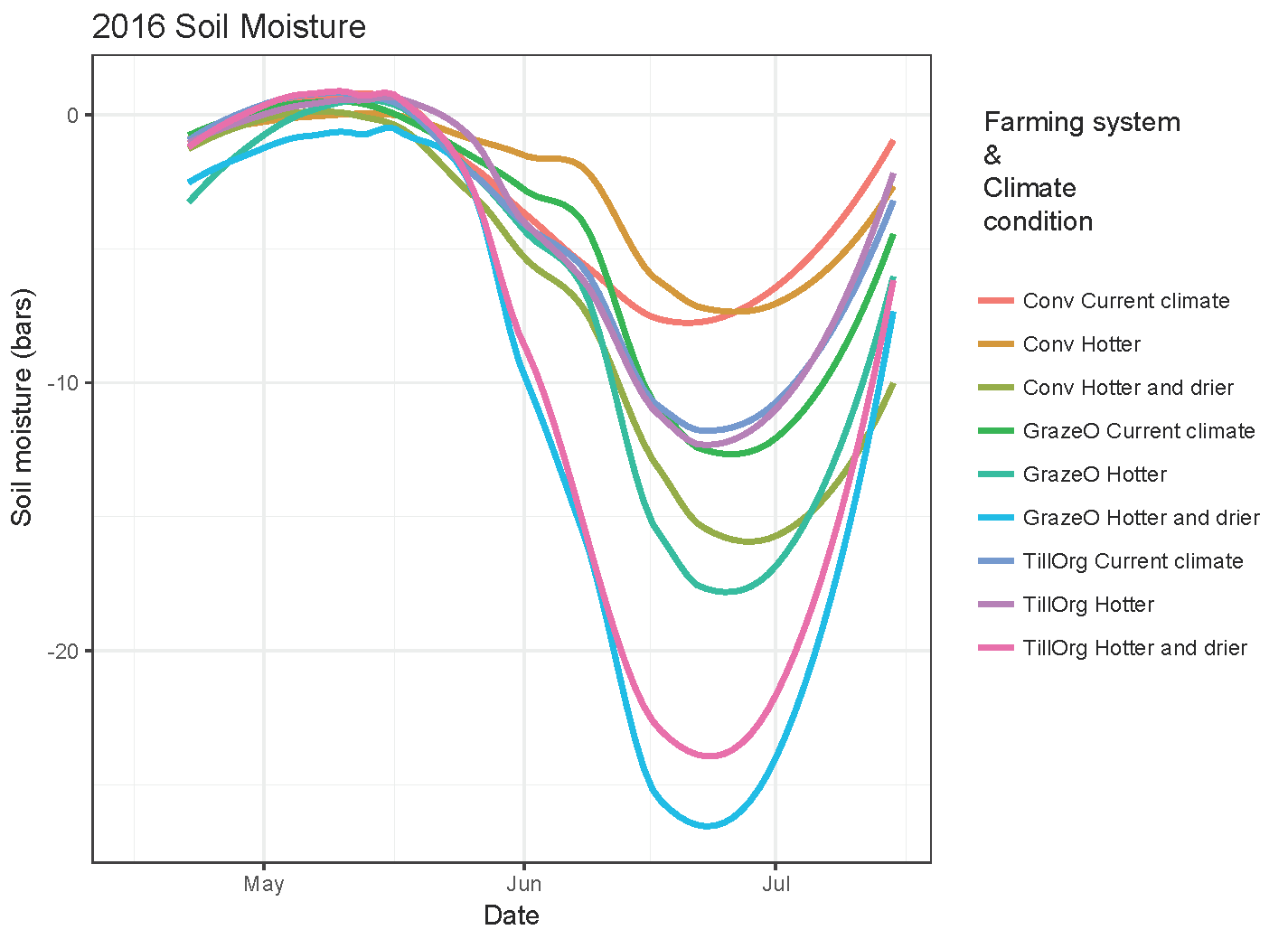

Supplement: FIG S2 [file mSphere.00340-20-sf002.tif]

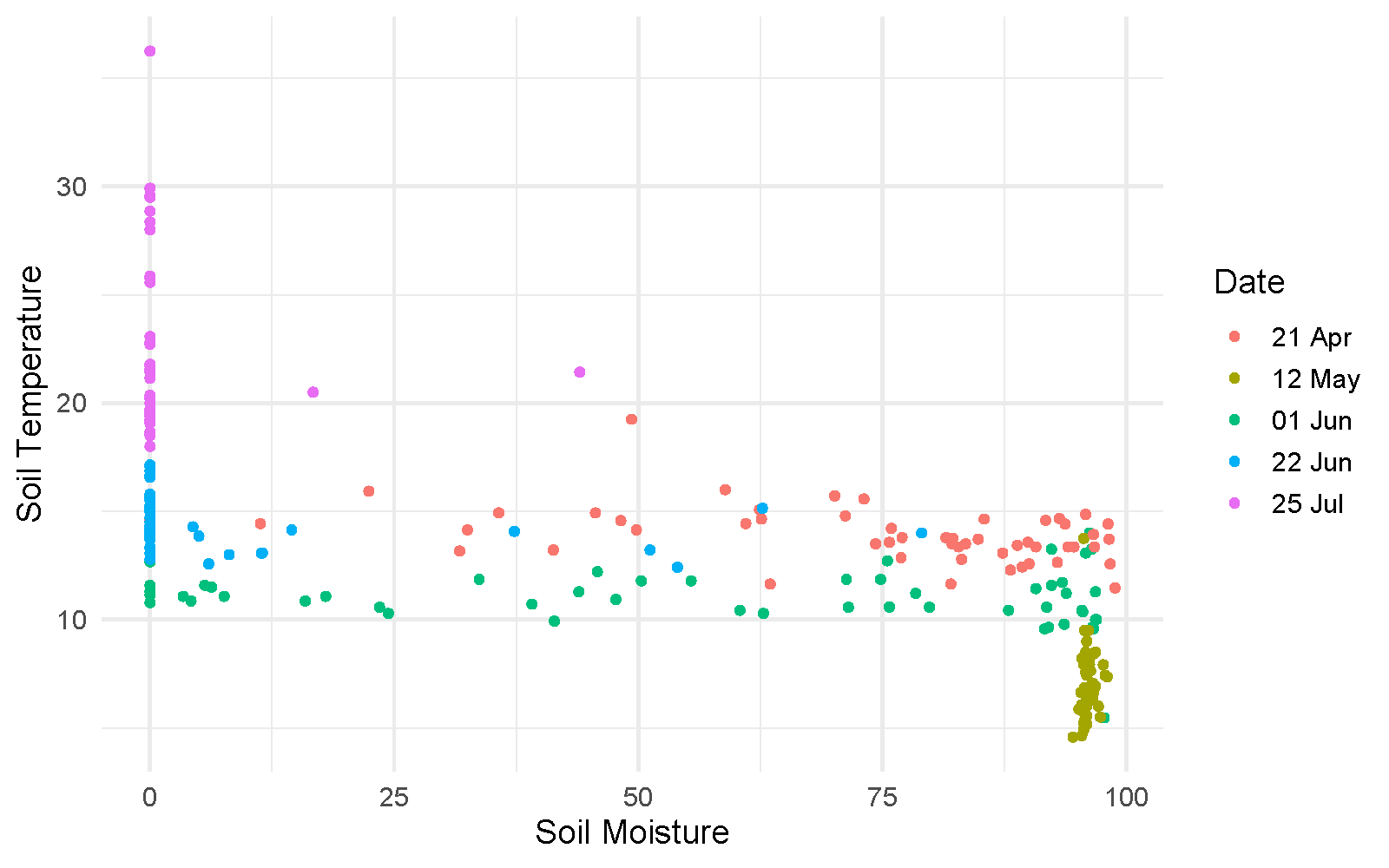

Supplement: FIG S3 [file mSphere.00340-20-sf003.tif]

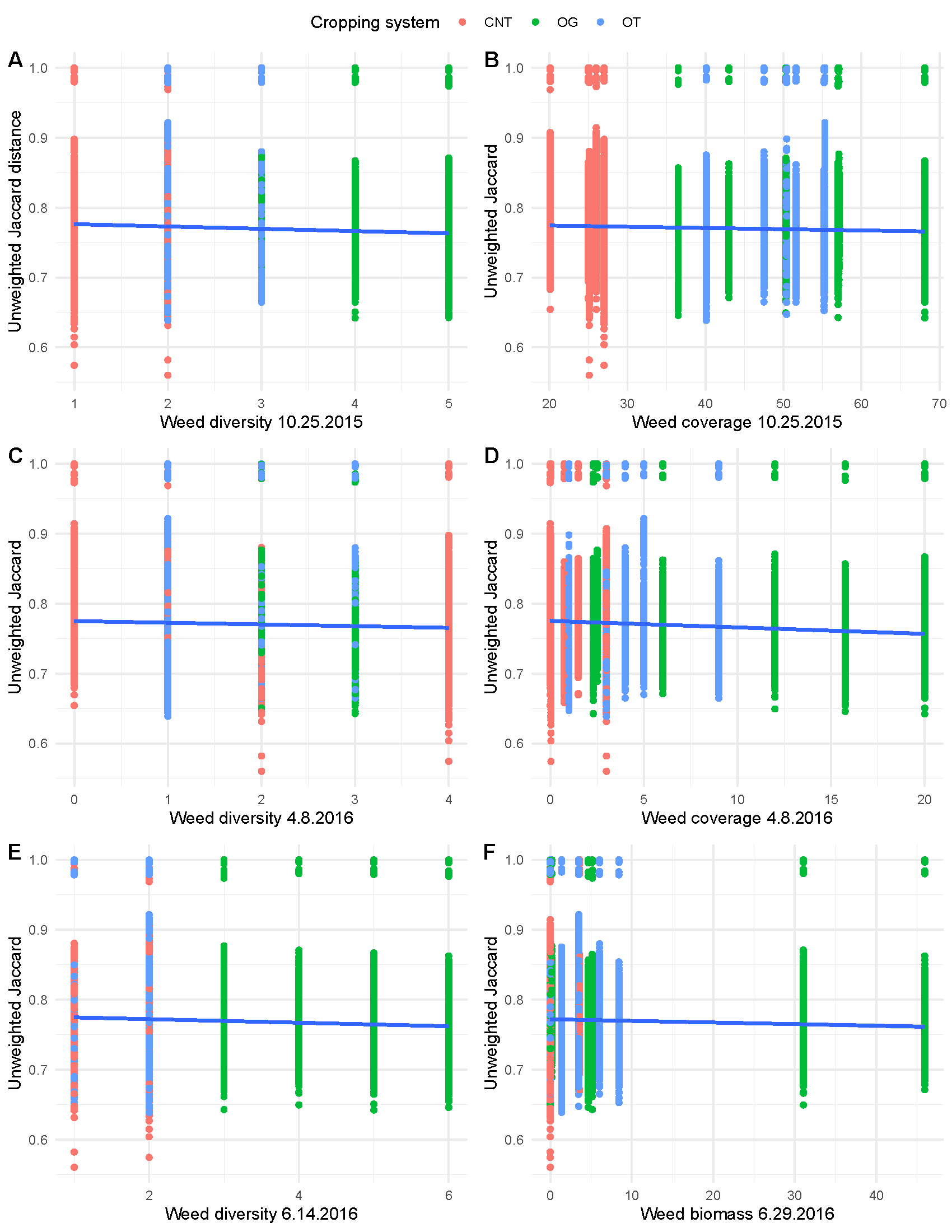

Supplement: FIG S4 [file mSphere.00340-20-sf004.tif]

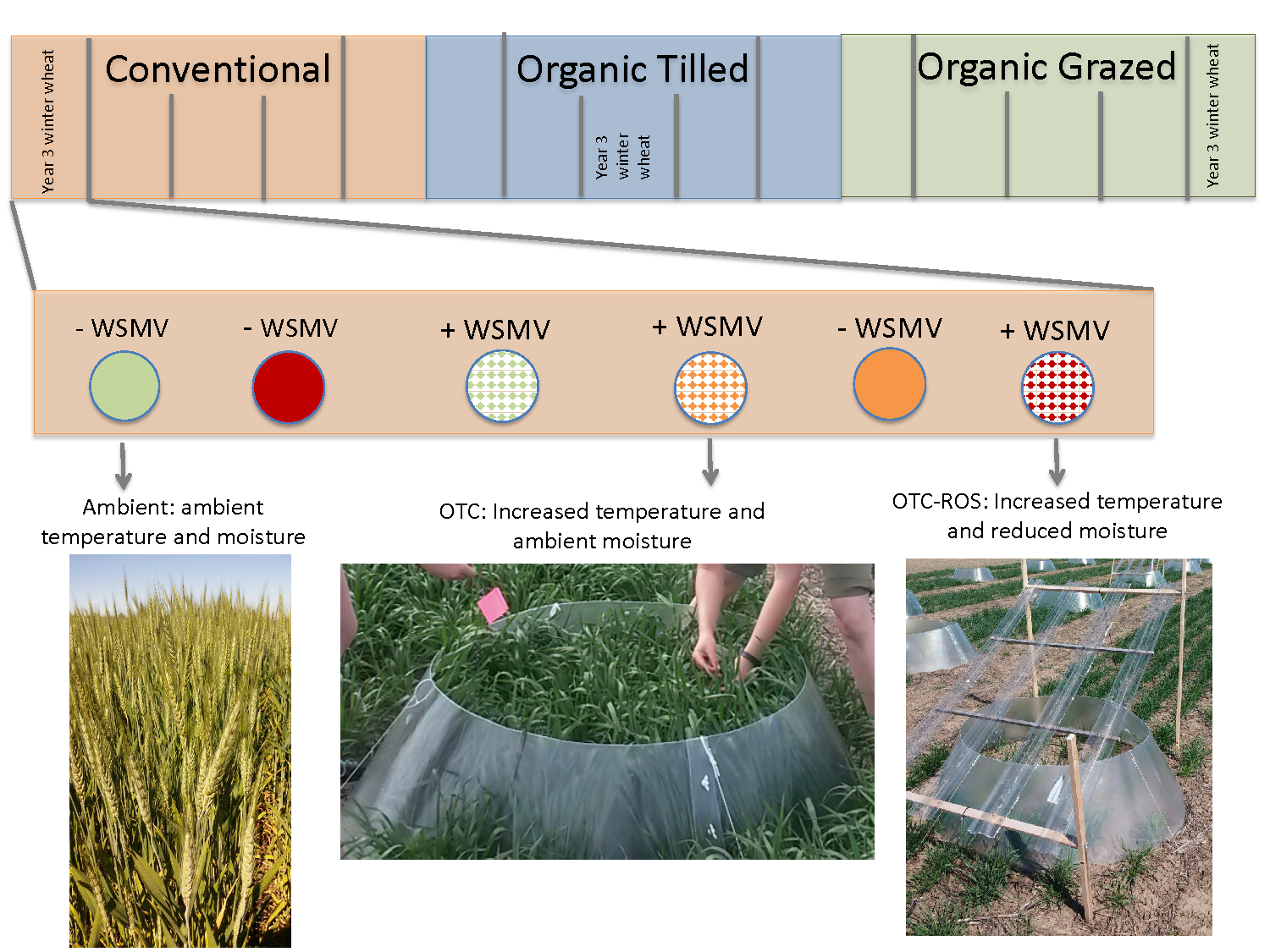

Supplement: FIG S5 [file mSphere.00340-20-sf005.tif]
