# Supplementary material for: Dryland Cropping Systems, Weed Communities, and Disease Status Modulate the Effect of Climate Conditions on Wheat Soil Bacterial Communities
Source: mSphere. 2020 Jul 15;5(4):e00340-20. doi: 10.1128/mSphere.00340-20 (PMC7364210; doi:10.1128/mSphere.00340-20)
Supplement: TABLE S1 [file mSphere.00340-20-st001.docx]

| **OTU** | **LDA** | ***p*** | **OTU** | **LDA** | ***p*** |
| --- | --- | --- | --- | --- | --- |
| **CNT** | | | | | |
| Acetobacteraceae | 2.51 | <0.001 | Gaiellales | 2.28 | 0.035 |
| Acidicaldus | 2.18 | <0.001 | Gaiellales | 2.39 | <0.001 |
| Acidicaldus | 2.44 | <0.001 | Gaiellales | 2.68 | <0.001 |
| Acidimicrobiales | 2.29 | <0.001 | Gammaproteobacteria | 2.10 | <0.001 |
| Acidobacteria Subgroup_6 | 2.28 | 0.008 | Gemmatimonadaceae | 2.17 | <0.001 |
| Acidobacteria Subgroup_6 | 2.35 | 0.026 | Gemmatimonadaceae | 2.24 | <0.001 |
| Acidobacteria Subgroup_6 | 2.47 | 0.002 | Gemmatimonas | 2.15 | <0.001 |
| Acidobacteria Subgroup_6 | 2.47 | 0.002 | Gemmatimonas | 2.32 | 0.002 |
| Acidobacteria Subgroup_6 | 2.47 | <0.001 | Haliangium | 2.03 | 0.019 |
| Acidobacteria Subgroup_6 | 2.90 | <0.001 | Haliangium | 2.22 | 0.001 |
| Actinobacteria | 2.23 | 0.001 | Haliangium | 2.35 | 0.001 |
| Actinobacteria | 2.34 | <0.001 | Iamia | 2.40 | 0.006 |
| Actinobacteria | 2.79 | 0.038 | Iamia | 2.43 | <0.001 |
| Actinobacteria MB-A2-108 | 2.22 | 0.006 | Iamia | 2.62 | <0.001 |
| Actinobacteria MB-A2-108 | 2.62 | <0.001 | Ilumatobacter | 2.81 | <0.001 |
| Altererythrobacter | 3.01 | <0.001 | Incertae_Sedis | 2.15 | 0.024 |
| Amycolatopsis | 2.26 | 0.016 | Kineosporiaceae | 2.47 | <0.001 |
| Anaerolineaceae | 2.34 | 0.015 | Kribbella | 2.36 | 0.020 |
| Angustibacter | 2.35 | 0.015 | Marmoricola | 2.48 | 0.021 |
| Arenimonas | 2.38 | <0.001 | Microbacteriaceae | 2.98 | <0.001 |
| Arenimonas | 2.41 | 0.004 | Micromonosporaceae | 2.23 | 0.036 |
| Betaproteobacteria | 2.42 | 0.016 | Micromonosporaceae | 2.27 | 0.024 |
| Betaproteobacteria Subgroup_6 | 2.16 | <0.001 | Myxococcales | 2.92 | 0.001 |
| Betaproteobacteria Subgroup_6 | 2.27 | <0.001 | Nakamurella | 2.67 | 0.001 |
| Betaproteobacteria Subgroup_6 | 2.33 | <0.001 | Nitrosomonadaceae | 2.50 | <0.001 |
| Betaproteobacteria Subgroup_6 | 2.49 | <0.001 | Nitrosospira | 3.05 | <0.001 |
| Bradyrhizobiaceae | 3.38 | 0.018 | Nitrospira | 2.26 | <0.001 |
| Bryobacter | 2.31 | <0.001 | Nocardioides | 2.07 | 0.007 |
| Candidatus_Microthrix | 2.29 | <0.001 | Nocardioides | 2.57 | <0.001 |
| Candidatus_Microthrix | 2.46 | <0.001 | Ohtaekwangia | 2.28 | <0.001 |
| Candidatus_Solibacter | 2.49 | <0.001 | OM27_clade | 2.42 | <0.001 |
| Catelliglobosispora | 2.48 | <0.001 | Oxalobacteraceae | 2.76 | 0.041 |
| Caulobacteraceae | 2.82 | 0.005 | Patulibacter | 2.76 | 0.004 |
| Chitinophaga | 2.07 | 0.010 | Phaselicystis | 2.38 | <0.001 |
| Chitinophagaceae | 2.10 | <0.001 | Phyllobacteriaceae | 2.66 | <0.001 |
| Chitinophagaceae | 2.64 | <0.001 | Proteobacteria | 2.33 | 0.032 |
| Chloroflexi KD4-96 | 2.14 | <0.001 | Proteobacteria | 2.71 | <0.001 |
| Chloroflexi KD4-96 | 2.57 | 0.001 | Pseudomonas | 2.82 | <0.001 |
| Chryseolinea | 2.77 | <0.001 | Pseudonocardia | 2.50 | <0.001 |
| Chthoniobacter | 2.23 | <0.001 | Ramlibacter | 2.21 | 0.029 |
| Chthoniobacterales DA101 | 2.16 | 0.028 | Reyranella | 2.37 | 0.009 |
| Comamonadaceae | 2.18 | <0.001 | Reyranella | 2.73 | <0.001 |
| Comamonadaceae | 2.23 | <0.001 | Rhizomicrobium | 2.77 | <0.001 |
| Comamonadaceae | 3.10 | <0.001 | Rhodospirillales DA111 | 2.30 | <0.001 |
| Cytophaga | 3.09 | <0.001 | Sandaracinaceae | 2.17 | 0.015 |
| Erythrobacteraceae | 2.66 | <0.001 | Sandaracinaceae | 2.25 | 0.004 |
| Ferruginibacter | 2.87 | 0.005 | Sandaracinaceae | 2.30 | <0.001 |
| Flavobacterium | 2.46 | <0.001 | Solirubrobacterales 480-2 | 2.43 | <0.001 |
| Flavobacterium | 2.52 | <0.001 | Solirubrobacterales 480-2 | 2.81 | 0.003 |
| Flavobacterium | 2.99 | 0.013 | Sphingomonadaceae | 2.13 | 0.003 |
| Flavobacterium | 3.69 | <0.001 | Streptomycetaceae | 2.88 | <0.001 |
| Gaiella | 2.26 | 0.006 | Subgroup_5 | 2.33 | <0.001 |
| Gaiella | 2.64 | 0.001 | Xanthomonadaceae | 2.75 | <0.001 |
| **OG** | | | | | |
| Acidimicrobiaceae CL500-29 | 2.19 | 0.002 | Iamia | 2.15 | <0.001 |
| Acidimicrobiales | 2.05 | 0.007 | Iamia | 2.19 | <0.001 |
| Acidimicrobiales | 2.27 | <0.001 | Iamia | 2.20 | 0.004 |
| Acidobacteria Subgroup_17 | 2.20 | <0.001 | Iamia | 2.24 | 0.029 |
| Acidobacteria Subgroup_6 | 2.18 | <0.001 | Iamia | 2.28 | <0.001 |
| Acidobacteria Subgroup_6 | 2.24 | <0.001 | Iamia | 2.53 | <0.001 |
| Acidobacteria Subgroup_6 | 2.24 | <0.001 | Ilumatobacter | 2.54 | <0.001 |
| Acidobacteria Subgroup_6 | 2.25 | <0.001 | Kineosporia | 2.51 | 0.005 |
| Acidobacteria Subgroup_6 | 2.26 | 0.011 | Lautropia | 2.16 | <0.001 |
| Acidobacteria Subgroup_6 | 2.32 | <0.001 | Marmoricola | 2.22 | <0.001 |
| Acidobacteria Subgroup_6 | 2.33 | <0.001 | Micrococcaceae | 2.30 | <0.001 |
| Acidobacteria Subgroup_6 | 2.35 | <0.001 | Micrococcaceae | 3.09 | <0.001 |
| Acidobacteria Subgroup_6 | 2.36 | <0.001 | Micromonosporaceae | 2.02 | <0.001 |
| Acidobacteria Subgroup_6 | 2.41 | 0.003 | Micromonosporaceae | 2.25 | <0.001 |
| Acidobacteria Subgroup_6 | 2.44 | <0.001 | Micromonosporaceae | 3.00 | <0.001 |
| Acidobacteria Subgroup_6 | 2.51 | 0.019 | Microvirga | 2.44 | <0.001 |
| Acidobacteria Subgroup_6 | 2.56 | <0.001 | Microvirga | 2.65 | <0.001 |
| Acidobacteria Subgroup_6 | 2.63 | <0.001 | Microvirga | 2.98 | <0.001 |
| Acidobacteria Subgroup_6 | 2.64 | <0.001 | Myxococcales | 2.11 | 0.005 |
| Acidobacteria Subgroup_6 | 2.66 | <0.001 | Myxococcales | 2.32 | <0.001 |
| Acidobacteria Subgroup_6 | 2.67 | <0.001 | Myxococcales | 2.70 | 0.011 |
| Acidobacteria Subgroup_6 | 2.74 | <0.001 | Myxococcales | 2.93 | <0.001 |
| Acidobacteria Subgroup_6 | 2.83 | <0.001 | Nitrosomonadaceae | 2.31 | 0.009 |
| Acidobacteria Subgroup_6 | 3.02 | <0.001 | Nitrosomonadaceae | 2.43 | <0.001 |
| Actinobacteria | 2.21 | 0.001 | Nocardioides | 2.10 | 0.014 |
| Actinobacteria | 2.25 | <0.001 | Nocardioides | 2.30 | 0.006 |
| Actinobacteria | 2.64 | 0.005 | Nocardioides | 2.49 | <0.001 |
| Actinobacteria | 2.75 | 0.021 | Nocardioides | 2.64 | <0.001 |
| Actinobacteria MB-A2-108 | 2.31 | <0.001 | Nocardioides | 2.68 | <0.001 |
| Actinobacteria MB-A2-108 | 2.47 | 0.005 | Nocardioides | 2.72 | <0.001 |
| Actinobacteria MB-A2-108 | 2.48 | 0.008 | Nocardioides | 2.74 | <0.001 |
| Actinobacteria RB41 | 2.26 | 0.026 | Opitutus | 2.02 | 0.003 |
| Adhaeribacter | 2.37 | 0.008 | Opitutus | 2.15 | <0.001 |
| Adhaeribacter | 2.38 | <0.001 | Oryzihumus | 2.86 | <0.001 |
| Adhaeribacter | 2.49 | <0.001 | Oxalobacteraceae | 2.09 | 0.027 |
| Aeromicrobium | 2.88 | <0.001 | Pedobacter | 2.67 | <0.001 |
| Agromyces | 2.14 | 0.004 | Pedomicrobium | 2.13 | 0.003 |
| Agromyces | 2.45 | <0.001 | Phaselicystis | 2.52 | 0.001 |
| Altererythrobacter | 2.61 | <0.001 | Proteobacteria | 2.09 | 0.040 |
| Altererythrobacter | 2.83 | <0.001 | Proteobacteria | 2.25 | <0.001 |
| Anaerolineaceae | 2.51 | <0.001 | Proteobacteria | 2.51 | <0.001 |
| Arthrobacter | 2.41 | <0.001 | Pseudolabrys | 2.63 | <0.001 |
| Asanoa | 2.32 | <0.001 | Pseudonocardia | 2.23 | 0.001 |
| Betaproteobacteria | 2.20 | 0.017 | Pseudonocardia | 2.45 | <0.001 |
| Betaproteobacteria Subgroup_6 | 2.23 | <0.001 | Pseudonocardiaceae | 2.32 | <0.001 |
| Betaproteobacteria TRA3-20 | 2.10 | 0.004 | Pseudospirillum | 2.06 | 0.008 |
| Betaproteobacteria TRA3-20 | 2.21 | <0.001 | Pseudospirillum | 2.12 | <0.001 |
| Betaproteobacteria TRA3-20 | 2.22 | <0.001 | Rhizobacter | 2.15 | 0.003 |
| Blastococcus | 2.92 | <0.001 | Rhizobiales | 2.11 | <0.001 |
| Blastococcus | 3.69 | 0.001 | Rhizobiales | 2.24 | 0.004 |
| Bosea | 2.10 | 0.010 | Rhizobiales | 2.30 | <0.001 |
| Bradyrhizobiaceae | 2.70 | <0.001 | Rhizobiales | 2.32 | <0.001 |
| Brevundimonas | 2.33 | <0.001 | Rhizobiales JG34-KF-361 | 2.35 | <0.001 |
| Bryobacter | 2.40 | <0.001 | Rhodococcus | 2.49 | <0.001 |
| Caenimonas | 2.21 | <0.001 | Rhodoplanes | 2.14 | <0.001 |
| Candidatus_Alysiosphaera | 2.21 | 0.005 | Rhodoplanes | 2.32 | <0.001 |
| Chitinophagaceae | 2.12 | 0.022 | Rubellimicrobium | 2.19 | <0.001 |
| Chitinophagaceae | 2.95 | <0.001 | Rubellimicrobium | 2.34 | <0.001 |
| Chloroflexi KD4-96 | 2.16 | <0.001 | Rubrobacter | 2.37 | <0.001 |
| Chloroflexi KD4-96 | 2.54 | <0.001 | Rubrobacter | 2.90 | <0.001 |
| Chthoniobacter | 2.20 | 0.008 | Rubrobacter | 2.92 | <0.001 |
| Chthoniobacter | 2.37 | 0.008 | Sanguibacter | 2.56 | <0.001 |
| Clostridium | 2.09 | 0.008 | Skermanella | 3.59 | <0.001 |
| Cytophagaceae | 2.71 | <0.001 | Solirubrobacter | 2.24 | 0.027 |
| Desulfurellaceae | 2.12 | <0.001 | Solirubrobacter | 2.37 | 0.004 |
| Devosia | 2.25 | <0.001 | Solirubrobacter | 2.38 | <0.001 |
| Ferruginibacter | 2.13 | 0.045 | Solirubrobacter | 2.45 | <0.001 |
| Flavisolibacter | 2.11 | 0.006 | Solirubrobacter | 2.59 | 0.042 |
| Flavisolibacter | 2.31 | <0.001 | Solirubrobacterales 0319-6M6 | 2.17 | <0.001 |
| Flavitalea | 2.16 | 0.020 | Solirubrobacterales 288-2 | 2.07 | 0.014 |
| Flavitalea | 2.18 | 0.005 | Solirubrobacterales 288-2 | 2.39 | <0.001 |
| Flavobacterium | 3.36 | <0.001 | Solirubrobacterales 288-2 | 2.73 | <0.001 |
| Fodinicola | 2.48 | 0.003 | Solirubrobacterales 288-2 | 2.87 | <0.001 |
| Gaiella | 2.33 | 0.017 | Solirubrobacterales 480-2 | 2.26 | <0.001 |
| Gaiella | 2.45 | <0.001 | Solirubrobacterales FFCH13075 | 2.63 | <0.001 |
| Gaiella | 2.69 | <0.001 | Sorangium | 2.12 | <0.001 |
| Gaiellales | 2.68 | <0.001 | Sphingomonadaceae | 2.47 | 0.040 |
| Gaiellales | 2.77 | <0.001 | Sphingomonas | 2.09 | 0.024 |
| Gemmatimonadaceae | 2.03 | 0.009 | Sphingomonas | 3.38 | <0.001 |
| Gemmatimonadaceae | 2.23 | <0.001 | Steroidobacter | 2.27 | <0.001 |
| Gemmatimonadaceae | 2.36 | <0.001 | Steroidobacter | 2.55 | <0.001 |
| Gemmatimonas | 2.13 | 0.001 | Thermomicrobia AKYG1722 | 2.07 | 0.016 |
| Gemmatimonas | 2.24 | <0.001 | Virgisporangium | 2.43 | <0.001 |
| Gitt-GS-136 | 2.89 | <0.001 | Xanthomonadaceae | 2.25 | <0.001 |
| Holophagae ABS-19 | 2.41 | <0.001 | Xanthomonadaceae | 2.44 | <0.001 |
| **OT** | | | | | |
| Acidobacteria Subgroup_6 | 2.14 | 0.004 | Gemmatimonas | 2.17 | 0.022 |
| Acidobacteria Subgroup_6 | 2.18 | 0.024 | Gemmatimonas | 2.31 | <0.001 |
| Acidobacteria Subgroup_6 | 2.22 | 0.009 | Gemmatimonas | 2.32 | 0.009 |
| Acidobacteria Subgroup_6 | 2.45 | <0.001 | Gemmatimonas | 2.35 | 0.016 |
| Acidobacteria Subgroup_6 | 2.49 | <0.001 | Gemmatimonas | 2.37 | 0.041 |
| Acidobacteria Subgroup_6 | 2.55 | <0.001 | Gemmatimonas | 2.49 | <0.001 |
| Acidobacteria Subgroup_6 | 2.58 | 0.003 | Gemmatimonas | 2.60 | <0.001 |
| Acidobacteriaceae Subgroup_1 | 2.53 | <0.001 | Gemmatimonas | 2.61 | <0.001 |
| Actinobacteria | 2.06 | 0.001 | Krasilnikovia | 2.75 | <0.001 |
| Actinobacteria | 2.24 | <0.001 | Massilia | 2.10 | <0.001 |
| Arenimonas | 2.48 | <0.001 | Massilia | 2.22 | <0.001 |
| Arthrobacter | 3.92 | <0.001 | Massilia | 2.44 | <0.001 |
| Bacillaceae | 2.74 | <0.001 | Massilia | 2.52 | <0.001 |
| Betaproteobacteria Subgroup_6 | 2.06 | <0.001 | Methylobacterium | 2.64 | <0.001 |
| Betaproteobacteria Subgroup_6 | 2.47 | <0.001 | Micromonosporaceae | 2.41 | <0.001 |
| Betaproteobacteria Subgroup_6 | 2.52 | <0.001 | Myxococcales | 2.46 | <0.001 |
| Blastocatella | 2.20 | 0.026 | Nocardioidaceae | 2.03 | 0.007 |
| Byssovorax | 2.23 | 0.027 | Nocardioides | 2.02 | 0.012 |
| Candidatus_Solibacter | 2.29 | <0.001 | Oryzihumus | 2.70 | <0.001 |
| Candidatus_Solibacter | 3.00 | <0.001 | Phenylobacterium | 2.39 | 0.002 |
| Chitinophagaceae | 2.31 | <0.001 | Pseudomonas | 2.39 | <0.001 |
| Chitinophagaceae | 2.36 | 0.010 | Rhizobiaceae | 2.42 | <0.001 |
| Chitinophagaceae | 2.67 | <0.001 | Rhizobium | 2.50 | <0.001 |
| Chthoniobacterales DA101 | 2.08 | 0.010 | Rhizomicrobium | 2.64 | <0.001 |
| Comamonadaceae | 2.09 | 0.002 | Rhodospirillales DA111 | 2.65 | 0.002 |
| Dactylosporangium | 2.58 | 0.003 | Roseiflexus | 2.32 | <0.001 |
| Devosia | 2.30 | 0.009 | Roseiflexus | 2.51 | <0.001 |
| Flavisolibacter | 2.30 | <0.001 | Segetibacter | 2.43 | <0.001 |
| Frankiales | 2.69 | 0.002 | Solirubrobacterales 288-2 | 2.34 | <0.001 |
| Gaiellales | 2.08 | 0.002 | Sphingomonadales | 2.46 | <0.001 |
| Gammaproteobacteria | 2.12 | 0.003 | Sphingomonas | 3.69 | <0.001 |
| Gemmatimonadaceae | 2.46 | <0.001 | Terrabacter | 2.33 | 0.019 |
| Gemmatimonadaceae | 2.57 | <0.001 | Thermomicrobia JG30-KF-CM45 | 2.58 | <0.001 |
| Gemmatimonadaceae | 2.61 | <0.001 | Xanthomonadaceae | 2.49 | <0.001 |
