# Supplementary material for: Dryland Cropping Systems, Weed Communities, and Disease Status Modulate the Effect of Climate Conditions on Wheat Soil Bacterial Communities
Source: mSphere. 2020 Jul 15;5(4):e00340-20. doi: 10.1128/mSphere.00340-20 (PMC7364210; doi:10.1128/mSphere.00340-20)
Supplement: TABLE S2 [file mSphere.00340-20-st002.docx]

| **Sample ID** | **Fraction of plot infected** | **Cropping System** | **Virus treatment** | **Climate** | **Plot** |
| --- | --- | --- | --- | --- | --- |
| OG_725_v_a_1021 | 0.85 | OG | WSMV | ambient | 1021 |
| OG_725_n_a_1022 | 0.35 | OG | none | ambient | 1022 |
| OG_725_v_o_1025 | 0.00 | OG | WSMV | OTC | 1025 |
| OG_725_n_o_1026 | 0.20 | OG | none | OTC | 1026 |
| OG_725_v_r_1029 | 0.15 | OG | WSMV | ROS | 1029 |
| OG_725_n_r_10210 | 0.15 | OG | none | ROS | 10210 |
| CC_725_v_a_1061 | 0.10 | CNT | WSMV | ambient | 1061 |
| CC_725_n_a_1062 | 0.00 | CNT | none | ambient | 1062 |
| CC_725_v_o_1065 | 0.15 | CNT | WSMV | OTC | 1065 |
| CC_725_n_o_1066 | 0.00 | CNT | none | OTC | 1066 |
| CC_725_v_r_1069 | 0.10 | CNT | WSMV | ROS | 1069 |
| CC_725_n_r_10610 | 0.20 | CNT | none | ROS | 10610 |
| OT_725_v_a_1141 | 0.00 | OT | WSMV | ambient | 1141 |
| OT_725_n_a_1141 | 0.00 | OT | none | ambient | 1142 |
| OT_725_v_o_1145 | 0.05 | OT | WSMV | OTC | 1145 |
| OT_725_n_o_1146 | 0.00 | OT | none | OTC | 1146 |
| OT_725_v_r_1149 | 0.05 | OT | WSMV | ROS | 1149 |
| OT_725_n_r_11410 | 0.00 | OT | none | ROS | 11410 |
| OT_725_v_a_2021 | 0.10 | OT | WSMV | ambient | 2021 |
| OT_725_n_a_2022 | 0.00 | OT | none | ambient | 2022 |
| OT_725_v_o_2025 | 0.00 | OT | WSMV | OTC | 2025 |
| OT_725_n_o_2026 | 0.00 | OT | none | OTC | 2026 |
| OT_725_v_r_2029 | 0.00 | OT | WSMV | ROS | 2029 |
| OT_725_n_r_20210 | 0.03 | OT | none | ROS | 20210 |
| OG_725_v_a_2091 | 0.50 | OG | WSMV | ambient | 2091 |
| OG_725_n_a_2092 | 0.00 | OG | none | ambient | 2092 |
| OG_725_v_o_2095 | 0.00 | OG | WSMV | OTC | 2095 |
| OG_725_n_o_2096 | 0.00 | OG | none | OTC | 2096 |
| OG_725_v_r_2099 | 0.10 | OG | WSMV | ROS | 2099 |
| OG_725_n_r_20910 | 0.00 | OG | none | ROS | 20910 |
| CC_725_v_a_2121 | 0.10 | CNT | WSMV | ambient | 2121 |
| CC_725_n_a_2122 | 0.00 | CNT | none | ambient | 2122 |
| CC_725_v_o_2125 | 0.15 | CNT | WSMV | OTC | 2125 |
| CC_725_n_o_2126 | 0.00 | CNT | none | OTC | 2126 |
| CC_725_v_r_2129 | 0.00 | CNT | WSMV | ROS | 2129 |
| CC_725_n_r_21210 | 0.00 | CNT | none | ROS | 21210 |
| CC_725_v_a_3051 | 0.00 | CNT | WSMV | ambient | 3051 |
| CC_725_n_a_3052 | 0.00 | CNT | none | ambient | 3052 |
| CC_725_v_o_3055 | 0.00 | CNT | WSMV | OTC | 3055 |
| CC_725_n_o_3056 | 0.00 | CNT | none | OTC | 3056 |
| CC_725_v_r_3059 | 0.00 | CNT | WSMV | ROS | 3059 |
| CC_725_n_r_30510 | 0.00 | CNT | none | ROS | 30510 |
| OT_725_v_a_3101 | 0.10 | OT | WSMV | ambient | 3101 |
| OT_725_n_a_3102 | 0.00 | OT | none | ambient | 3102 |
| OT_725_v_o_3105 | 0.00 | OT | WSMV | OTC | 3105 |
| OT_725_n_o_3106 | 0.00 | OT | none | OTC | 3106 |
| OT_725_v_r_3109 | 0.10 | OT | WSMV | ROS | 3109 |
| OT_725_n_r_31010 | 0.15 | OT | none | ROS | 31010 |
| OG_725_v_a_3151 | 0.05 | OG | WSMV | ambient | 3151 |
| OG_725_n_a_3152 | 0.05 | OG | none | ambient | 3152 |
| OG_725_v_o_3155 | 0.00 | OG | WSMV | OTC | 3155 |
| OG_725_n_o_3156 | 0.00 | OG | none | OTC | 3156 |
| OG_725_v_r_3159 | 0.00 | OG | WSMV | ROS | 3159 |
| OG_725_n_r_31510 | 0.00 | OG | none | ROS | 31510 |
| controls | 0.00 | na | na | na | bfr |
| controls | 0.00 | na | na | na | bfr1 |
| controls | 0.00 | na | na | na | bfr2 |
| controls | 0.00 | na | na | na | bfr3 |
| controls | 0.00 | na | na | na | bfr4 |
| controls | 0.00 | na | na | na | bfr5 |
| positive control | 1.00 | na | na | na | cont |
| negative control | 0.00 | na | na | na | ncont |
| other | 0.20 | na | na | na | other |
