# Supplementary material for: Dryland Cropping Systems, Weed Communities, and Disease Status Modulate the Effect of Climate Conditions on Wheat Soil Bacterial Communities
Source: mSphere. 2020 Jul 15;5(4):e00340-20. doi: 10.1128/mSphere.00340-20 (PMC7364210; doi:10.1128/mSphere.00340-20)
Supplement: TABLE S3 [file mSphere.00340-20-st003.docx]

|  | **April** | **May** | **June** | **July** |
| --- | --- | --- | --- | --- |
| 2016 Max °C | 25.5 | 28.3 | 35 | 36.7 |
| 2016 Min °C | 6.1 | 4.4 | 16.1 | 18.9 |
| 1981 - 2010 Max °C | 13.6 | 18.6 | 22.7 | 28.2 |
| 1981 - 2010 Min °C | -1.1 | 3.3 | 6.7 | 9.8 |
|  |  |  |  |  |
| 2016 Mean Precipitation (mm)^1^ | 53 | 53 | 55 | 22 |
| 1981 - 2010 Mean Precipitation (mm)^2^ | 45 | 71 | 71 | 36 |

1 Data from Montana.gov Station Reports, 2016

2 Data from U.S. Climate Data, PRISM Climate Group
