# Supplementary material for: Dryland Cropping Systems, Weed Communities, and Disease Status Modulate the Effect of Climate Conditions on Wheat Soil Bacterial Communities
Source: mSphere. 2020 Jul 15;5(4):e00340-20. doi: 10.1128/mSphere.00340-20 (PMC7364210; doi:10.1128/mSphere.00340-20)
Supplement: TABLE S4 [file mSphere.00340-20-st004.docx]

| **Cropping system** | **Climate** | **Virus** | **pH** | **OM (%)** | **Nitrate-N ppm** | **P-Olsen ppm** | **K ppm** |
| --- | --- | --- | --- | --- | --- | --- | --- |
| CNT | Ambient | none | 6.07 ^a^ (0.58) | 5.23 ^b^ (0.5) | 3.50 (1.00) | 42.33 (7.57) | 408 (91.03) |
| CNT | Hotter | none | 6.5 ^cd^ (0.66) | 5.17 ^e^ (0.38) | 25.67 ^fg^ (3.25) | 44.67 (5.51) | 434 (58.23) |
| CNT | Hotter/ drier | none | 6.2 ^h^ (0.36) | 5.20 ^i^ (0.40) | 31.67 ^jk^ (4.01) | 45.33 (8.14) | 375.33 ^l^ (77.53) |
| CNT | Ambient | WSMV | 6.47 (0.75) | 5.50 (0.95) | 5.67 (3.25) | 47.33 (9.45) | 401.33 (66.94) |
| CNT | Hotter | WSMV | 6.30 ^cmn^ (0.56) | 5.20 (0.70) | 23.67 (14.22) | 47.67 (8.08) | 402 (100.41) |
| CNT | Hotter/ drier | WSMV | 5.87 ^o^ (0.49) | 5.60 ^i^ (0.50) | 33.67 ^pq^ (10.21) | 49.33 (11.06) | 424.67 ^l^ (68.97) |
| OT | Ambient | none | 6.77 (1.06) | 6.57 (0.64) | 3.50 (3.28) | 55.0 (30.32) | 514 (278.12) |
| OT | Hotter | none | 6.33 (0.45) | 6.37 (0.83) | 4.83 ^f^ (3.82) | 50.33 (24.38) | 500 (258.53) |
| OT | Hotter/ drier | none | 6.63 (0.85) | 6.03 (0.32) | 2.83 ^j^ (1.53) | 48.0 (14.53) | 504.33 (170.02) |
| OT | Ambient | WSMV | 6.97 (0.55) | 5.97 (0.68) | 3.67 (2.57) | 54.33 (14.98) | 521 (107.22) |
| OT | Hotter | WSMV | 6.73 ^m^ (0.65) | 6.23 (0.49) | 2.33 (0.76) | 46.67 (18.48) | 461.67 (212.55) |
| OT | Hotter/ drier | WSMV | 6.30 ^r^ (0.36) | 6.23 (0.40) | 6.33 ^p^ (3.51) | 54.33 (10.69) | 554.67 (87.51) |
| OG | Ambient | none | 7.00 ^a^ (0.36) | 5.97 ^b^ (0.15) | 4.50 (4.77) | 47.33 (4.93) | 530 (278.68) |
| OG | Hotter | none | 6.97 ^d^ (0.55) | 6.00 ^e^ (0.61) | 3.17 ^g^ (2.08) | 55.33 (11.06) | 491.33 (135.09) |
| OG | Hotter/ drier | none | 7.30 ^hs^ (0.56) | 5.40 (0.35) | 2.83 ^k^ (1.15) | 47.67 (3.79) | 419.67 (153.96) |
| OG | Ambient | WSMV | 7.27 (0.35) | 5.80 (0.61) | 3.33 (2.75) | 54.33 (20.21) | 470 (132.92) |
| OG | Hotter | WSMV | 7.50 ^n^ (0.36) | 5.63 (0.51) | 2.17 (0.29) | 52.33 (19.6) | 525.33 (201.17) |
| OG | Hotter/ drier | WSMV | 6.73 ^ors^ (0.61) | 5.73 (0.4) | 2.67 ^q^ (1.04) | 45.0 (1.73) | 486.33 (104.94) |
